# A potent and selective small molecule inhibitor of myoferlin attenuates colorectal cancer progression

**DOI:** 10.1002/ctm2.289

**Published:** 2021-02-07

**Authors:** Yuan He, Weiqiong Kan, Yunqi Li, Yun Hao, Anling Huang, Haijun Gu, Minna Wang, Qingqing Wang, Jinlian Chen, Zhenliang Sun, Mingyao Liu, Yihua Chen, Zhengfang Yi

**Affiliations:** ^1^ East China Normal University and Shanghai Fengxian District Central Hospital Joint Center for Translational Medicine Shanghai Key Laboratory of Regulatory Biology Institute of Biomedical Sciences and School of Life Sciences East China Normal University Shanghai 200241 P.R. China; ^2^ Joint Center for Translational Medicine Southern Medical University Affiliated Fengxian Hospital Shanghai 201499 P.R. China

**Keywords:** colorectal cancer, myoferlin, rab, small molecule inhibitor

## Abstract

As a pivotal vesicular trafficking protein, Myoferlin (MYOF) has become an attractive target for cancer therapy. However, the roles of MYOF in colorectal cancer invasion remain enigmatic, and MYOF‐targeted therapy in this malignancy has not been explored. In the present study, we provided the first functional evidence that MYOF promoted the cell invasion of colorectal cancer. Furthermore, we identified a novel small molecule inhibitor of MYOF (named YQ456) that showed high binding affinity to MYOF (K_D_ = 37 nM) and excellent anti‐invasion capability (IC_50_ = 110 nM). YQ456 was reported for the first time to interfere with the interactions between MYOF and Ras‐associated binding (Rab) proteins at low nanomolar levels. This interference disrupted several vesicle trafficking processes, including lysosomal degradation, exosome secretion, and mitochondrial dynamics. Further, YQ456 exhibited excellent inhibitory effects on the growth and invasiveness of colorectal cancer. As the first attempt, the anticancer efficacy of YQ456 in the patient‐derived xenograft (PDX) mouse model indicated that targeting MYOF may serve as a novel and practical therapeutic approach for colorectal cancer.

## Graphical abstract

- A novel and potent small molecule MYOF inhibitor (YQ456) exhibits anti‐growth and anti‐invasive effects against colorectal cancer with low toxicity.
- We first reveal the positive correlation between the expression level of MYOF and the metastatic potential of colorectal cancer.
- YQ456 interrupts the interactions between MYOF and Rabs (Rab7 and Rab32).

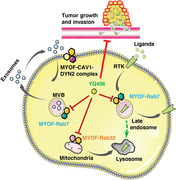

**Abbreviations**
ATCC: American Type Culture Collection
BLI: biolayer interferometry
CAV1: caveolin 1
CLSM: confocal laser scanning microscopy
co‐IP: co‐immunoprecipitation
DAPI: 4′,6‐diamidino‐2‐phenylindole
DMSO: dimethyl sulfoxide
DRP1: dynamin‐related protein 1
DYN2: dynamin 2
DYSF: dysferlin
EGFR: epidermal growth factor receptor
EMT: epithelial‐mesenchymal transition
ESI: electrospray ionization
H&E: hematoxylin and eosin
HPLC: high‐resolution mass spectrum
HRMS: high‐resolution mass spectrum
HUVEC: human umbilical vein endothelial cell
IC_50_: half inhibition concentration
IF: immunofluorescence
IHC: immunohistochemistry
IVIS: in vivo imaging system
LDA: limiting dilution analysis
MMP: mitochondrial membrane potential
MYOF: myoferlin
MYOF‐C2D: C2D domain of MYOF
NMR: nuclear magnetic resonance
OCR: oxygen consumption rate
OIS: oncogene‐induced senescence
OXPHOS: oxidative phosphorylation
PBS: phosphate buffer saline
PDX: patient‐derived xenograft
Rab: Ras‐associated binding
ROS: reactive oxygen species
RTK: receptor tyrosine kinase
S637: serine‐637
sgMYOF: MYOF sgRNA
sgNC: negative control sgRNA
SPR: surface plasmon resonance
VEGF: vascular endothelial growth factor
VEGFR: vascular endothelial growth factor receptor

## 1 INTRODUCTION

In 2018, colorectal cancer ranks third in incidence, but second in terms of cancer mortality worldwide.^1^ More than 90% of colorectal cancer deaths are attributed to the significant metastatic potential of this malignancy.^2^ Despite the development of chemotherapy drugs, metastatic colorectal cancer remains an enormous health burden. Clinically approved drugs, including vascular endothelial growth factor (VEGF) inhibitors and epidermal growth factor receptor (EGFR) inhibitors, are often accompanied by drug resistance and tumor recurrence.^3^, ^4^ Therefore, it is urgent to identify innovative and effective therapeutic drugs for the clinical therapy of metastatic colorectal cancer. Vesicle trafficking in cancer cells is responsible for the loss of cell polarity and the acquisition of invasive and metastatic properties.^5^ Therefore, targeting dysregulated vesicle trafficking system is an attractive approach for cancer therapy.^5^ Recently, vesicle trafficking‐related protein myoferlin (MYOF) has drawn growing interest as a potential anticancer target. Previous evidence revealed that overexpression of MYOF is positively correlated with the poor survival rates of patients with breast cancer,^6^ pancreatic cancer,^7^ and colon cancer.^8^ The structure of MYOF is characterized by multiple functional C2 domains, which are related to the proliferative and metastatic behaviors of cancers.^9^, ^10^ For example, MYOF enhances the stability of vascular endothelial growth factor receptor (VEGFR), thus facilitating the angiogenesis process in clear cell renal cell carcinoma.^11^ MYOF is a crucial mediator of EGFR in maintaining normal signaling transmission in hepatocellular carcinoma.^12^ Moreover, MYOF, as a novel exosomal component, is involved in exosome biology.^13^ MYOF depletion inhibits exosome fusion and cargo transport into target cells.^14^ Furthermore, MYOF is essential to regulate oxidative phosphorylation (OXPHOS) activity and mitochondrial dynamics, which are related to cell survival and drug resistance in pancreatic ductal adenocarcinoma.^15^ A previous work illustrated that MYOF inhibits cell proliferation in colorectal cancer by modulating OXPHOS.^8^ However, the precise mechanisms of MYOF in the cell invasiveness of colorectal cancer remain uncertain.

Activated small GTPases Ras‐associated binding (Rab) proteins are recruited to various intracellular membranes to mediate the vesicular trafficking processes in cancer cells.^16^ The Rab family plays pivotal roles in the malignant transformation and aggressive invasion of cancer cells by regulating the functions of organelles, such as endosome, lysosome, and mitochondria.^17^ Among them, Rab7 is an essential organizer of the late endosomal/lysosomal proteolytic system, which recruits specific effectors to regulate cargo transport, receptor recycling, and mitophagy.^18^ Therefore, the excessive activation of Rab7 results in the aberrant distribution of signaling receptors and the abnormal degradation of damaged organelles, which ultimately leads to cell proliferation, cell invasion, and poor prognosis of cancers.^19^ Notably, Rab32 is the only Rab GTPase, which localizes to mitochondrial membrane to regulate mitochondrial morphology and mitochondrial dynamics.^20^ Activated dynamin‐related protein 1 (DRP1) is recruited from cytoplasm to mitochondrial membrane, thus promoting mitochondrial fission process. A previous study proposed that Rab32 knockdown reduces the phosphorylation of Drp1 at serine‐637 (S637). This dephosphorylation can induce mitochondrial collapse and mitochondrial fragmentation by activating Drp1.^21^ In addition, the mitochondrion damage caused by Rab32 depletion leads to impaired oxidative respiratory chain and energy metabolism, thus promoting the accumulation of reactive oxygen species (ROS) and inducing cell apoptosis.^22^

Therefore, targeting vesicular trafficking modulates multiple cancer cell behaviors, which may provide an effective therapeutic method for colorectal cancer. Although MYOF and Rabs have similar functions in regulating vesicular trafficking, their exact relationships remain unknown. In our present work, we have investigated the crucial roles of MYOF in colorectal cancer cell invasiveness. Our further research reveals the previously unrecognized relationships between MYOF and Rab7, and between MYOF and Rab32. Remarkably, we have identified a novel MYOF inhibitor YQ456, which exhibits anti‐cancer activities by modulating RTK signaling pathways, endocytosis/exocytosis, and mitochondrial metabolism. Moreover, we have explored the therapeutic efficacy of YQ456 in clinical colorectal cancer tissues. Therefore, YQ456 targeting MYOF provides a promising therapeutic approach for colorectal cancer.

## 2 MATERIALS AND METHODS

### 2.1 Cell lines and animals

HCT116, LoVo, SW620, SW480, HT29, HCT15, HCT8, LS174T (human colorectal cancer cell lines), NCM460 (normal colonic epithelial cell line), and CT26 (mouse colon cancer cell line) were purchased from American Type Culture Collection (ATCC). Human umbilical vein endothelial cell (HUVEC) was obtained from ScienCell Research Laboratories. HCT116, LoVo, SW620, SW480, HT29, HCT15, HCT8, and CT26 were cultured in RPMI‐1640 medium. SW620, SW480, and LS174T were cultured in DMEM medium. HUVEC was cultured with ECM medium. The cell medium was supplemented with 10% FBS, 1% penicillin and streptomycin. Two patient‐derived cell lines were established from colorectal cancer patients and cultured in DMEM‐F12 medium according to the previous literature.^23^ BALB/c nude mice and BALB/c mice were purchased from National Rodent Laboratory Animal Resources, Shanghai Branch of China.

### 2.2 Chemicals

The synthesis scheme and identification of the YQ456 were showed in Figure S1‐3.

The key intermediate methyl 3‐(3‐ethyl‐5‐(4‐methoxyphenyl)‐1H‐1,2,4‐triazol‐1‐yl) benzoate was obtained according to the reported procedure.^24^ To a solution of methyl 3‐(3‐ethyl‐5‐(4‐methoxyphenyl)‐1*H*‐1,2,4‐triazol‐1‐yl) benzoate (337 mg, 1 mmol) in methanol (6 mL) were added dropwise LiOH·H_2_O (168 mg, 4 mmol) dissolved in water (1.5 mL) in an ice bath under Nitrogen protection, and then reacted at 25°C overnight. Methanol was removed by evaporation under reduced pressure. Hydrochloric acid (3 M) was added to adjust the pH to 3, then extracted twice with ethyl acetate, washed the organic phase with water and saturated brine, dried with Na_2_SO_4_, and concentrated to obtain the crude product 3‐(3‐ethyl‐5‐(4‐methoxyphenyl)‐1H‐1,2,4‐triazol‐1‐yl) benzoic acid (yield: 90%).

To a solution of the 3‐(5‐(4‐methoxyphenyl)‐3‐ethyl‐1*H*‐1,2,4‐triazol‐1‐yl) benzoic acid (65 mg, 0.2 mmol), EDC·HCl (50 mg, 0.26 mmol) and HOBt (30 mg, 0.22 mmol) in DMF (2 mL) was added 4‐phenylbutylamine (45 mg, 0.3 mmol) under ice bath, then reacted at room temperature for 3 h. After extraction with ethyl acetate and purification by column chromatography, the target product 3‐(3‐ethyl‐5‐(4‐methoxyphenyl)‐1H‐1,2,4‐triazol‐1‐yl)‐N‐(4‐phenylbutyl) benzamide (named YQ456) was obtained as white solid (yield: 63%). Compound stock solutions were prepared in dimethyl sulfoxide (DMSO) at a concentration of 50 mM and stored at –20°C.

### 2.3 Transwell invasion assay

Cell invasion ability was evaluated by transwell invasion assay. Colorectal cancer cells (1 × 10^5^ per well) were resuspended in the culture medium containing various concentrations of tested compounds. Then, cancer cells were seeded in transwell chambers (8 μm pore size) (Millipore) coated with 10% Matrigel (Corning). After 36 h, invasive cells on the lower side of chambers were fixed with 4% paraformaldehyde for 20 min, followed by staining with 0.35% crystal violet for 6 min. The cells were washed with water and air‐dried, followed by taking images using an inverted microscope.

### 2.4 Surface plasmon resonance (SPR) assay

The SPR assay was performed using a Biacore T200 instrument (GE). According to the standard amine‐coupling method, the C2D domain of MYOF (MYOF‐C2D) peptide diluted in sodium acetate buffer (pH 4.5) was immobilized on a CM5 sensor chip. Various concentrations of tested compounds were diluted in phosphate buffer saline (PBS) for affinity assessment. After the double subtraction of blank and reference background, binding affinities were obtained based on the 1:1 Langmuir binding model of BIAevaluation software.

### 2.5 Cell viability assay

Colorectal cancer cells were seeded in 96‐well plates. Adherent cells were incubated with various concentrations of compounds. HCT116 cells (3 × 10^3^ per well) and Lovo cells (3 × 10^3^ per well) were treated for 1 ‐ 5 days, two patient‐derived cell lines (6 × 10^3^ per well) were treated for 72 h. Cell viability was measured according to the instruction of an MTS assay kit (Promega). The half inhibition concentration (IC_50_) value was calculated using GraphPad Prism 8 software.

### 2.6 Colony formation assay

Colorectal cancer cells (2 × 10^3^ per well) were incubated with YQ456 for 10 days. After being fixed with 4% paraformaldehyde for 20 min, cell colonies were stained with 0.35% crystal violet for 25 min. Then, cell colonies were washed with water and air‐dried, followed by taking images using an inverted microscope.

### 2.7 Cell apoptosis assay

Colorectal cancer cells were seeded in 6 cm culture dishes. After 12 h, cells at 50% confluence were treated with various concentrations of YQ456 for 72 h. After washing with PBS, cells were resuspended in the binding buffer, then operated according to the instruction of the Apoptosis Detection Kit (BD Biosciences). Cell apoptosis was detected by BD FACSCalibur flow cytometer (BD Biosciences).

### 2.8 The construction of MYOF knockout cell lines

The lentiviral vector carrying Cas9 and sgRNAs targeting MYOF was constructed using the lentiCRISPR‐v2 vector (Addgene, #52961). According to the instructions of Lipofectamine 2000 reagent (Invitrogen), HEK293T cells in a 10 cm culture dish (70% confluence) were cotransfected with the following plasmids: the lentiviral vector of MYOF (10 μg), psPAX2 plasmid (10 μg), and pMD2.G plasmid (5 μg). After 72 h post‐transfection, cell supernatant was harvested and the final volume was concentrated to 2 mL. The sequences of negative sgRNA and sgRNAs against MYOF used in human‐derived cell lines (HCT116 and LoVo) and murine‐derived cell line (CT26) were described in Table S1.

The cell culture medium (2 mL) containing viral solution (1 mL) and polybrene (10 μg/mL) was added into the indicated cancer cells at 50% confluence in a six‐well plate. After incubation for 12 h, the culture medium was renewed and cells were cultured for 36 h. Stably transfected cells were selected by puromycin (1 μg/mL) for 15 days. Monoclonal cell lines were obtained by single‐cell picking, followed by the detection of MYOF expression using western blot assay.

### 2.9 Immunoblotting analysis

The cell lysate was lysed in sample buffer (150 mM NaCl, 0.5 mM EDTA, 1% NP‐40, 10% Glycerol, 50 mM Tris, protease inhibitors, and phosphatase inhibitors, pH 8.0). The lysate was fractionated in SDS/polyacrylamide gel and transferred to the nitrocellulose filter. The blots were incubated with indicated antibodies, followed by fluorescent‐labeled secondary antibodies, and quantified using a laser scanning imaging system (Li‐Cor, Odyssey). Primary antibodies are listed in Table S2.

### 2.10 The liver metastasis mouse model of CT26‐Luc cells

For splenic inoculation, an incision located below the left rib cage of mouse was made to exteriorize the spleen (the right side of mouse image). CT26‐Luc cells (1 × 10^6^ per mouse) stably expressing luciferase reporter gene were injected into the spleens of male BALB/c mice (6‐week‐old). After the inoculation, each mouse was intraperitoneally injected with 3 mg d‐luciferin potassium salt (Synchem, 360222), followed by isoflurane anesthesia for 2 min. About 8 min later, the fluorescence signal in liver (the left side of mouse image) was detected by *in vivo* imaging system (IVIS). According to the fluorescence values on day 0, a total of 48 mice were equally divided into 6 groups (n = 8): control group (DMSO), regorafenib group (25 mg/kg), YQ456 groups (25 and 50 mg/kg), negative control sgRNA (sgNC) group (DMSO), and MYOF sgRNA (sgMYOF) group (DMSO). All mice were intraperitoneally injected with 50 μL corresponding drug or DMSO daily for 20 days, except mice in the regorafenib group were treated by intragastric administration. The corresponding fluorescence signals in livers were monitored every 10 days. Kaplan‐Meier survival curve was made for survival analysis.

### 2.11 The xenograft mouse model of HCT116 cells and patient‐derived xenograft mouse model

HCT116 cells (3 × 10^6^ per mouse) were subcutaneously implanted into male BALB/c nude mice (6‐week‐old) to establish the growth mouse model. After the tumor grew to 200 mm^3^, a total of 35 mice were divided into 5 groups on average (n = 7): control group (DMSO), regorafenib group (25 mg/kg), YQ456 groups (25 and 50 mg/kg), and sgMYOF group (DMSO). The body weights and tumor volumes of mice were measured every 5 days.

The tumor tissues derived from two colorectal cancer patients were subcutaneously implanted into male BALB/c nude mice (6‐week‐old) to establish the patient‐derived xenograft (PDX) mouse model. After two consecutive subcutaneous passages of tumor tissues, about six tumor pieces (3 mm^3^) per mouse were subcutaneously implanted into BALB/c nude mice. Finally, a total of 31 mice (12 tumor tissues from patient #1 and 19 tumor tissues from patient #2, respectively) were successfully modeled. When the tumor grew to 100 mm^3^, mice were divided into 4 groups: control group (DMSO, n = 6), regorafenib group (25 mg/kg, n = 7), and YQ456 groups (25 and 50 mg/kg, n = 7). The body weights and tumor volumes of mice were measured every 4 days.

During the two experiments, all mice were intraperitoneally injected with 50 μL drug or DMSO daily, except mice in the regorafenib group were treated by intragastric administration. The tumor volumes of mice were calculated by the following formula: length × width × width × 0.52. When the tumor volumes of mice reached 2000 mm^3^, mice were euthanized with carbon dioxide due to ethical consideration.

### 2.12 Immunohistochemistry (IHC) analysis

Tumor samples were fixed in 4% paraformaldehyde overnight, then dehydrated in ethanol series. Tumor samples were embedded into the paraffin and then cut into 5 μm sections. The sections were incubated with indicated antibodies in IHC assay. All samples were stained with hematoxylin and eosin (H&E) to indicate the nucleus and cytoplasm, respectively. Primary antibodies were listed in Table S2.

### 2.13 Receptor tyrosine kinase (RTK) array

The phosphorylated RTK array was performed by a human phosphorylated RTK array kit (R&D Systems). After cell attachment, HCT116 cells in 10 cm dishes were cultured in the medium with or without 400 nM YQ456. After incubation for 24 h, cells were harvested and lysed in the lysis buffer for 40 min on ice. According to the manufacturer's instruction of this kit, the membrane coated with capture antibodies was incubated with cell lysate. After washing with PBS, the blot was incubated with the anti‐phosphotyrosine‐horseradish peroxidase antibody and detected by the Chemi Reagent Mix.

### 2.14 Co‐immunoprecipitation (co‐IP) assay

The co‐IP experiment was performed utilizing a co‐IP kit (Thermo Scientific). HEK293T cells and HCT116 cells were transfected with or without indicated plasmids, respectively. After 24 h post‐transfection, cells were treated with YQ456 for 24 h. The cell lysate was prepared in IP buffer (150 mM NaCl, 0.1% Triton X‐100, 50 mM Tris‐HCl, 1 mM EDTA, pH 7.4). After centrifugation at 11000 g for 25 min, cell lysate supernatants were incubated with corresponding antibodies overnight. The cell lysate was incubated with pre‐cleared protein A/G for 4 h. Following centrifugation and washing, the precipitates were subjected to western blot assay. All co‐IP experiments were performed at 4°C.

### 2.15 Immunofluorescence (IF) assay

After treatment with YQ456 for 24 h, colorectal cancer cells were fixed with 4% paraformaldehyde for 25 min. Then cells were permeabilized with 0.3% Triton X‐100 in PBS for 4 min. Cells were blocked with 2% bovine serum albumin for 30 min and incubated with specific antibodies overnight at 4 ℃. In addition, cells were incubated with indicated secondary antibodies for 1.5 h at room temperature. Cellular nuclei were counterstained with 4′,6‐diamidino‐2‐phenylindole (DAPI) for 3 min. Fluorescence signals were detected using a laser confocal microscope (Leica). Primary antibodies were listed in Table S2.

### 2.16 Sphere formation assay

HCT116 cells (300 per well) were seeded in a 96‐well flat‐bottom ultra‐low attachment plate (Corning). The plate was loaded with serum‐free DMEM/F12 medium containing 0.4% bovine serum albumin, 20 ng/mL of fibroblast growth factor and epidermal growth factor, 2% B27, 5 μg/mL of insulin, and various concentrations of YQ456. The culture medium was changed every 3 days. After incubation for 9 days, cell spheres were observed and photographed by the inverted microscope (Olympus).

### 2.17 Total exosome isolation

Exosomes were isolated using a Total Exosome Isolation Reagent Kit (Invitrogen). After the attachment of HCT116 cells, culture mediums in 10 cm dishes were replaced by serum‐free cell culture medium with or without YQ456. After incubation for 24 h, cells were harvested and centrifuged at 2500 × g for 25 min. The supernatant was added to the exosome precipitant and incubated overnight at 4°C. The mixture was centrifuged at 11 000 × g for 1 h at 4°C. Exosome pellets were resuspended in 50 μL PBS.

### 2.18 Extracellular flux analysis

The oxygen consumption rate (OCR) of HCT116 cells was determined using a Seahorse XF96 Extracellular Flux Analyzer (Agilent). HCT116 cells (2 × 10^4^ per well) were seeded in XFp mini‐plates (Agilent) and allowed to attach overnight. Cells were pre‐treated with YQ456 for 5 h and successively stressed with oligomycin (2 μM), FCCP (1 μM), and rotenone/antimycin A (0.5 μM) mix. Mitochondrial OCR was measured following the manufacturer's instructions. Results were normalized based on cell number per well.

### 2.19 Mitochondrial membrane potential assay

Mitochondrial membrane potential was determined following the manufacturer's instructions (Beyotime). Cells in 6‐well plates were treated with YQ456 for 24 h. Then cells were washed with PBS and incubated with JC‐1 probe in 1× dye working buffer for 20 min at 37°C. After washing with cleaning buffer, JC‐1 monomers and JC‐1 aggregates were observed under a fluorescence microscope to indicate mitochondrial membrane potential.

### 2.20 Intracellular ROS production assay

Intracellular ROS levels were measured by a Reactive Oxygen Species Assay Kit (Beyotime). YQ456‐treated cells were incubated with the serum‐free medium containing DCFH‐DA for 20 min at 37°C. Then cells were washed with serum‐free medium, the cell fluorescence was observed and photographed by a fluorescence microscope.

### 2.21 siRNA‐mediated knockdown of genes

HCT116 cells at 50% confluence in six‐well plates were transfected with 100 nM siRNA targeting Rab7 or Rab32 using 4 μL Lipofectamine 2000 reagent (Invitrogen) in serum‐free medium. Six hours later, cells were switched to fresh complete medium and incubated for 36 h. The knockdown efficiency of siRNAs was measured by western blot. The sequences of negative siRNA and siRNAs against Rab7 and Rab32 were described in Table S1.

### 2.22 Statistical analysis

Data are presented as mean ± SD. Statistical analyses were performed with GraphPad Prism Software version 8.0. Student's t‐test (two‐tailed) was used for comparison between two groups. Comparisons of multiple groups were performed with the one‐way or two‐way ANOVA. Differences among multiple groups at different time points were analyzed using the two‐way repeated measurement ANOVA. Kaplan–Meier survival curves were analyzed using a log‐rank test. *P*‐value < .05 was considered statistically significant. Each assay was repeated at least three independent times except for animal experiments.

## 3 RESULTS

### 3.1 The screening and identification of MYOF inhibitor

**FIGURE 1:**
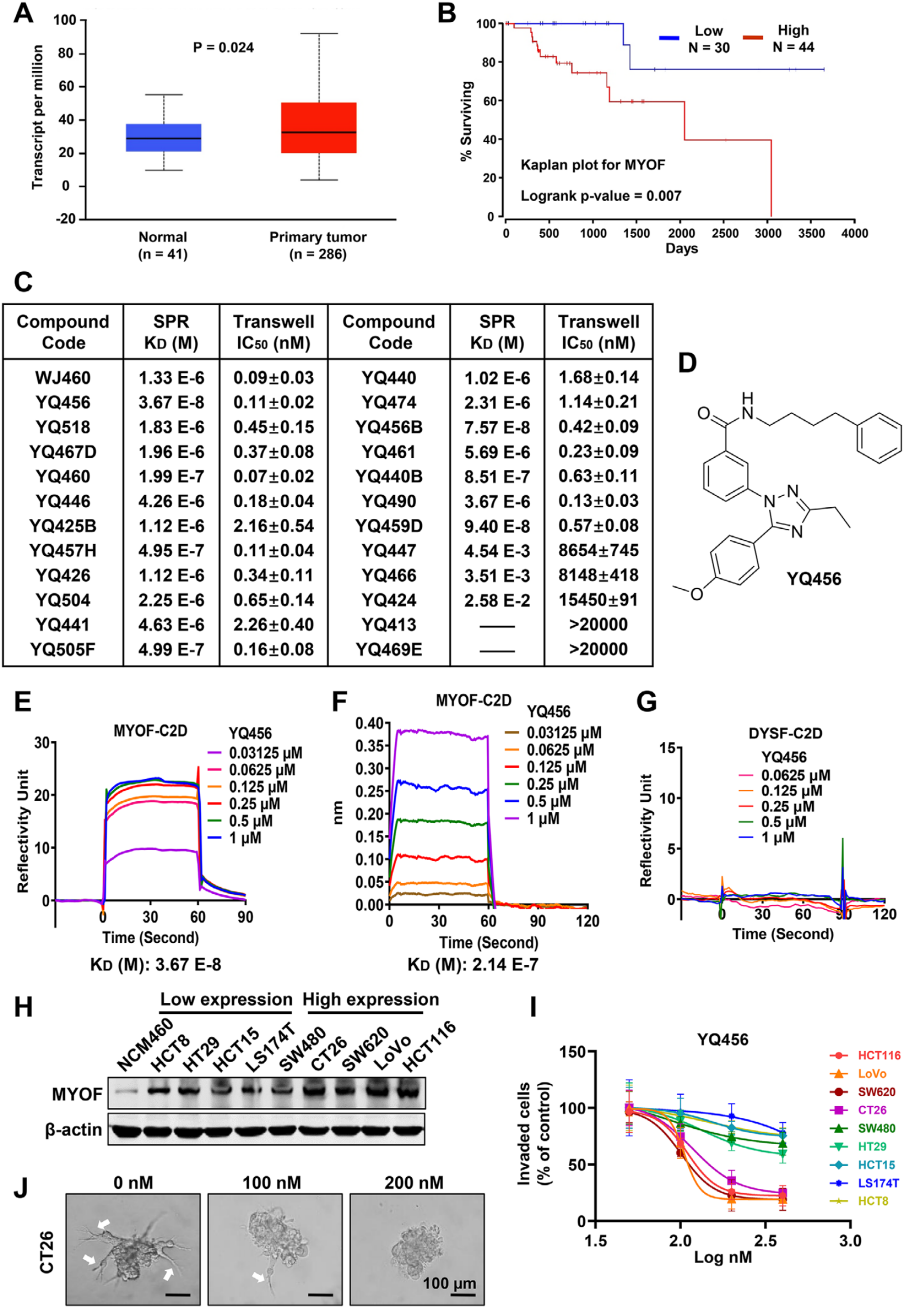
YQ456 targets MYOF to suppress colorectal cancer invasion *in vitro*. A, Data from the UALCAN database showed the expression levels of MYOF in colorectal cancer tissues and normal tissues (*P* = .024). B, Data from the OncoLnc database showed that patients with high MYOF expression have a poor prognosis compared with patients with low MYOF expression (*P* = .007). C, The binding affinities versus anti‐invasion activities of 2,3‐diaryl‐4‐thiazolidinone derivative analogs. D, The chemical structure of YQ456. E, The binding affinity between YQ456 and MYOF‐C2D domain in SPR analysis. F, The binding affinity between YQ456 and MYOF‐C2D domain in BLI assay. G, The binding affinity between YQ456 and DYSF‐C2D domain in SPR analysis. H, The expression levels of MYOF in colorectal cancer cells and normal colon epithelial cells. I, The anti‐invasion effect of YQ456 on various colorectal cancer cell lines was evaluated by transwell invasion analysis. J, The representative images of YQ456‐treated CT26 cells in 3D Matrigel invasion assay. White arrows indicate stretched pseudopodia. Scale bars, 100 μm. Data are presented as mean ± SD

Although the role of MYOF in colorectal cancer metastasis has not been revealed, studies have explored that MYOF promotes cancer metastasis in several cancer types. Therefore, we hypothesized that targeting MYOF may provide a novel therapeutic approach for metastatic colorectal cancer. Evidence from the UALCAN and The Human Protein Atlas databases presented that the expression of MYOF in colorectal cancer tissues was higher than that in normal tissues (Figure 1A; Figure S4A). As shown in Figure S4B and Figure 1B, the expression level of MYOF was positively correlated with the metastatic capacity of colorectal cancer in the UALCAN database and the poor clinical survival of colorectal cancer patients in the OncoLnc database (lower percentile: 7, upper percentile: 10). These findings were consistent with the previous report that high MYOF expression is associated with low survival in colorectal cancer patients.^8^ In our previous study, we have identified that a small molecule compound (named WJ460) prevents breast cancer metastasis by targeting MYOF‐C2D domain.^6^ Subsequently, approximately 300 novel small molecule compounds were synthesized based on the analysis of structure‐activity relationship to improve the binding affinities and targeting specificities of WJ460 analogs. First, the transwell invasion assay was carried out to evaluate the effects of the derivatives of WJ460, and those analogs with potent anti‐invasion efficacies were tested by SPR assay using the purified MYOF‐C2D domain. Among the investigated derivatives, 19 compounds exhibited strong binding affinities to MYOF‐C2D domain in SPR assay and nanomole‐level anti‐invasion activities in transwell invasion assay (Figure 1C). Subsequently, the binding affinities, biological activities, and chemical structures of all compounds were comprehensively analyzed. Ultimately, 3‐(3‐ethyl‐5‐(4‐methoxyphenyl)‐1H‐1,2,4‐triazol‐1‐yl)‐N‐(4‐phenylbutyl)benzamide (named YQ456) was chosen for follow‐up studies (Figure 1D) due to its highest binding affinity to MYOF‐C2D domain (K_D_ = 37 nM) and excellent anti‐invasion capability (IC_50_ = 110 nM). Interestingly, the binding affinity of YQ456 was 36 times higher than that of WJ460, implying that YQ456 had a higher binding specificity to MYOF‐C2D domain (Figures 1C–1E). Besides, the binding affinity of YQ456 to MYOF‐C2D domain was further confirmed by biolayer interferometry (BLI) assay (K_D_ = 214 nM) (Figure 1F). Dysferlin (DYSF), another member of the Ferlin family, was selected as a negative control due to its highest sequence similarity (56%) with MYOF. However, no binding affinity was detected between YQ456 and DYSF‐C2D domain, implying the binding between YQ456 and MYOF was highly specific (Figure 1G). In summary, YQ456 displays a high binding affinity to MYOF with the promising anti‐invasion activity.

### 3.2 YQ456 hinders cell invasion of colorectal cancer

Colorectal cancer cell lines showed higher expression levels of MYOF than NCM460 cell line (Human normal colon epithelial cell line) (Figure 1H). Remarkably, the anti‐invasion effect of YQ456 increased in parallel with the expression level of MYOF (Figure 1I; Figure S4C), partly supporting that the anti‐invasion effect of YQ456 was dependent on the expression level of MYOF. As shown by 3D matrigel assay, the morphology of CT26 cells was characterized by stretched pseudopodia (indicated by white arrows). In contrast, cells treated with YQ456 showed smaller and shrunk multicellular clusters (Figure 1J). Epithelial‐mesenchymal transition (EMT) plays a critical role in cancer cell invasion by allowing polarized epithelial cells to break through the basement membrane barrier.^25^ MYOF depletion reversed the invasive morphology of cancer cells, as evidenced by reduced mesenchymal representative proteins (Fibronectin and Vimentin) and increased principal epithelial cell adhesion proteins (E‐cadherin and ZO‐1) (Figure S4D). Similarly, the reversal effects of EMT were observed in YQ456‐treated cells with or without EGF stimulation (Figure S4E,F). Additionly, the above findings were consistent with the IF results (Figure S4G and H). In summary, YQ456 restrains the invasion and infiltration of colorectal cancer cells by targeting MYOF.

### 3.3 YQ456 exhibits anti‐proliferative effect in colorectal cancer cells *in vitro*

**FIGURE 2:**
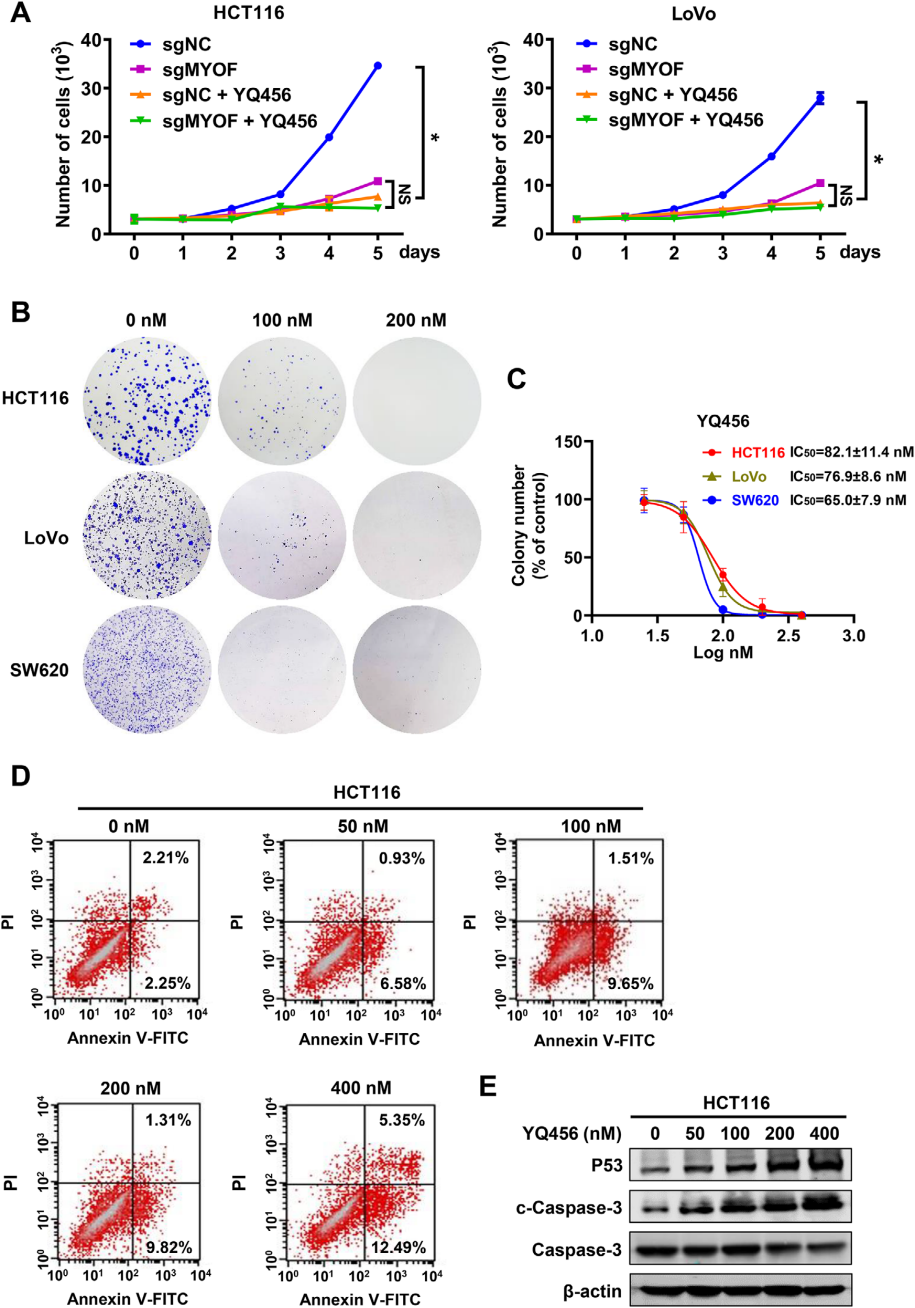
YQ456 restrains the proliferation of colorectal cancer cells. A, YQ456 inhibits HCT116 and LoVo cell proliferation in MTS assay. B, The representative clone formation images of HCT116, LoVo, and SW620 cells with YQ456 treatment. C, The statistical diagram of clone formation assay. D, The pro‐apoptosis activity of YQ456 in HCT116 cells. E, The regulation of YQ456 on apoptotic signaling pathway in HCT116 cells. Data are presented as mean ± SD. *P < .05; NS, not significant

Several independent assays were performed to evaluate the anti‐proliferative potencies of YQ456. As described in Figure 2A, the MTS analysis illustrated that both YQ456 treatment and MYOF depletion hindered the proliferation of HCT116 cells and LoVo cells. However, YQ456 displayed no significant inhibition on the cell proliferation of sgMYOF cells. Moreover, YQ456 robustly inhibited the clone formation of colorectal cancer cells (Figures 2B,C). The Annexin V/PI apoptosis assay presented that YQ456 increased the apoptosis of HCT116 cells (Figure 2D). The cell apoptosis was confirmed by the increased expression of pro‐apoptotic proteins, such as cleaved caspase‐3 and p53 (Figure 2E). To sum up, YQ456 exerts obvious inhibitory effects on the cell proliferation of colorectal cancer by targeting MYOF.

### 3.4 YQ456 prevents colorectal cancer growth *in vivo*

**FIGURE 3:**
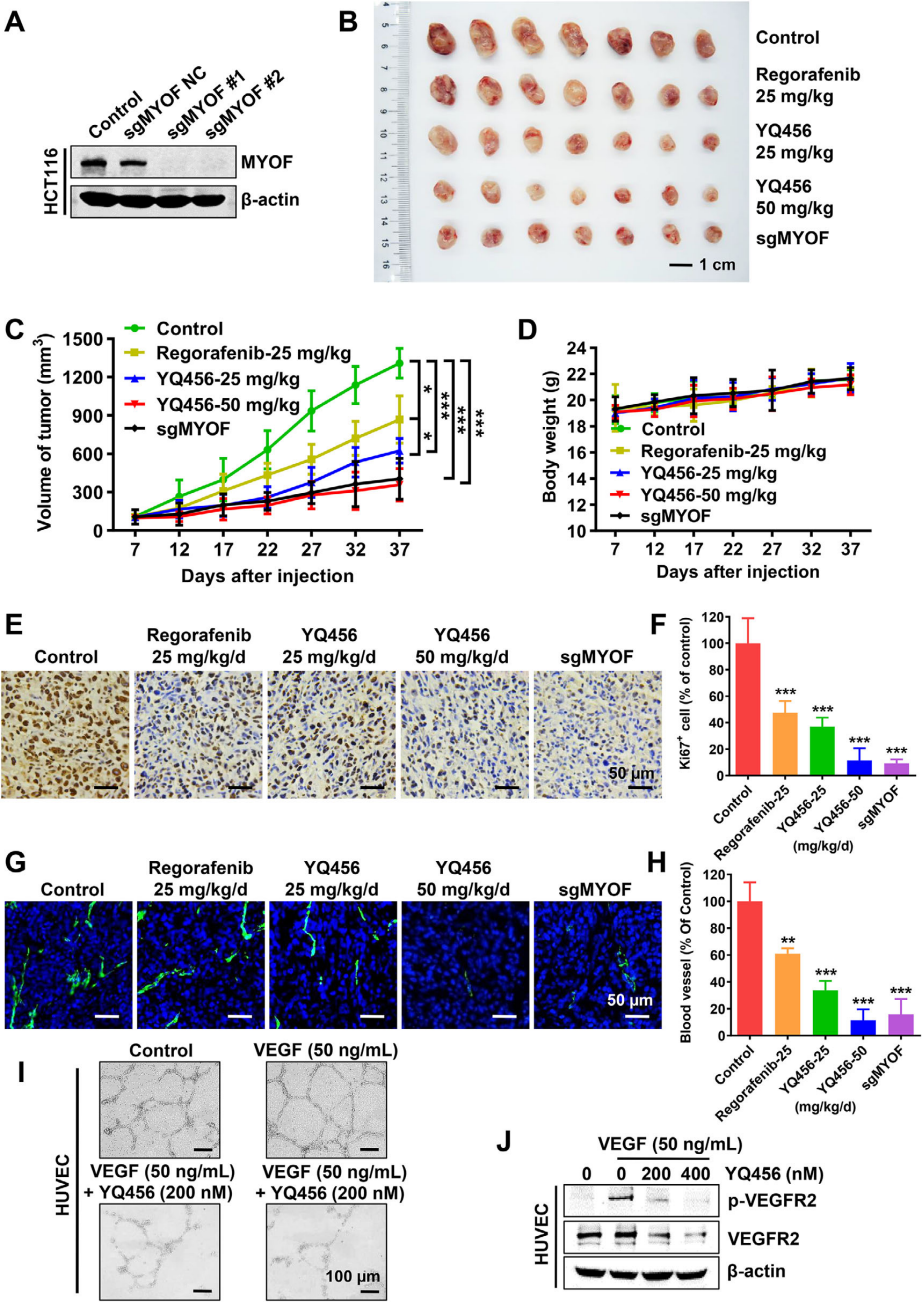
YQ456 prevents colorectal cancer growth in a subcutaneous xenograft mouse model. A, The expression of MYOF in HCT116 cells with sgMYOF treatment. B, The tumor morphology image of mice in each group. Scale bars, 1 cm. C, The statistical analysis of tumor volume in each group. D, The body weights of mice in each group. E, The IHC staining analysis of Ki‐67 expression (brown) in tumors. Nuclei were counterstained with DAPI (blue). Scale bars, 50 μm. F, The quantification of Ki67 immunostaining. G, The fluorescent signal of endothelial marker CD31 (green) in tumor tissues. Nuclei were counterstained with DAPI (blue). Scale bars, 50 mm. H, The statistical graph of CD31‐labeled tumor vessels. I, YQ456 prevented the VEGF‐induced capillary‐like tube formation of HUVECs. J, YQ456 suppressed the VEGF‐induced phosphorylation of VEGFR2 in HUVECs. Data are presented as mean ± SD. *P < .05; **P < .01; ***P < .001

A subcutaneous xenograft mouse model was used to test the *in vivo* antitumor effects of YQ456. MYOF was silenced in HCT116 cells, and the sgMYOF #1 cell line was selected for follow‐up studies (Figure 3A). In addition, RTK inhibitor regorafenib served as a positive control. The tumor volumes of mice in the drug‐treated and sgMYOF groups were significantly decreased compared with that in the control group (Figures 3B,C). Notably, YQ456 showed a higher inhibitory effect on tumor growth than regorafenib at the same concentration (Figures 3B,C). Furthermore, no weight losses and drug‐induced deaths of mice were observed in the YQ456 groups (Figure 3D), implying the low toxic side effects of YQ456.

Several assays were then performed to explore the anti‐proliferative mechanism of YQ456. The IHC assay revealed the expression of Ki67 (a cell proliferation marker) in the YQ456 and sgMYOF groups was dramatically decreased compared with that in the control group (Figure 3E,F). Previous work reported that MYOF promotes tumor growth by modulating VEGF‐induced angiogenesis.^11^ Blood vessel density (shown by CD31) in the YQ456 and sgMYOF groups was markedly decreased compared with that in the control group (Figures 3G,H). In addition, YQ456 interfered with the complete network structure formation of HUVECs in tubular formation assay, implying the impairment of angiogenesis process *in vivo* (Figure 3I). Furthermore, YQ456 suppressed the expression of VEGFR2 and VEGF‐induced p‐VEGFR2 in VEGF/VEGFR2 pathway (Figure 3J). The anti‐growth and anti‐invasion activities of YQ456 were validated by western blot assay of tumor tissues (Figure S5A). Moreover, both YQ456 treatment and sgMYOF treatment caused no observable histological damage to major organs (Figure S5B). Together, YQ456 effectively prevents colorectal cancer growth *in vivo* without apparent toxicity.

### 3.5 YQ456 attenuates colorectal cancer metastasis *in vivo*

**FIGURE 4:**
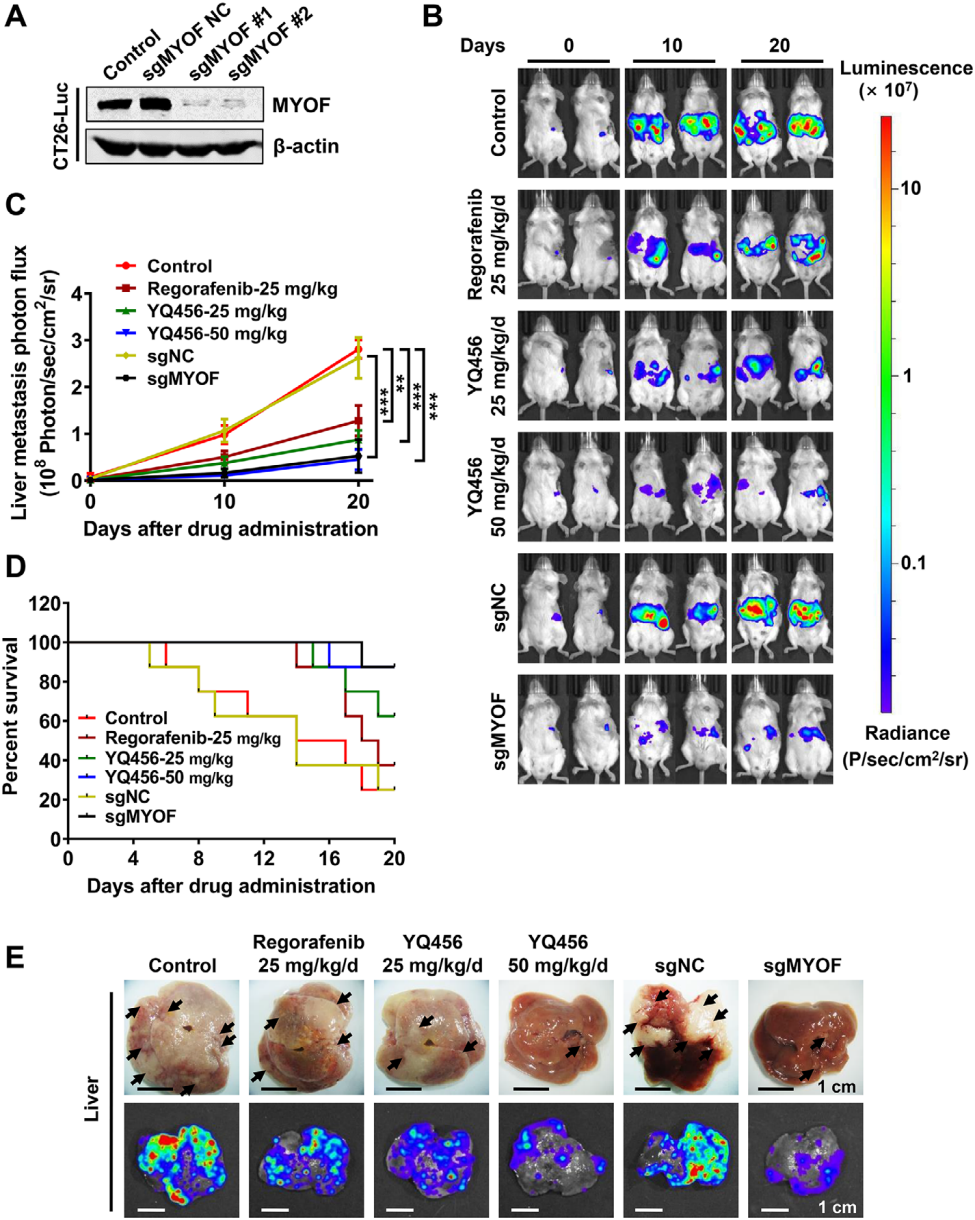
YQ456 suppresses the liver metastasis of colorectal cancer. A,The expression of MYOF in CT26‐Luc cells with sgRNA knockdown. B, The fluorescence of CT26‐Luc cells was detected by IVIS every 10 days to reflect the liver metastasis from the spleen. C, The photoluminescence statistics of CT26‐Luc cells in the livers of mice. D, The survival curve of mice. E, The photographs and fluorescence images of livers were taken to detect the liver metastasis of colorectal cancer. Scale bars, 1 cm. Data are presented as mean ± SD. **P < .01; ***P < .001

The anti‐metastatic activity of YQ456 was explored using a liver metastasis mouse model constructed by implanting CT26‐Luc cells into the spleen of BALB/c mice (Figures 4A,B). The fluorescence signals in livers (the left side of mouse images in Figure 4B) were detected by IVIS. The anti‐metastatic activity of YQ456 was significantly higher than that of regorafenib at the same dose (Figure 4C). Besides, CT26‐Luc cells with sgMYOF treatment showed reduced metastatic potential compared with parent CT26‐Luc cells, implying that MYOF played a pivotal role in colorectal cancer metastasis (Figures 4B,C). Given the liver metastasis of colorectal cancer accounts for the majority of patient mortality, the Kaplan‐Meier curve analysis was performed. The survival times of mice in the YQ456 groups was longer than that in the control and regorafenib groups (Figure 4D). Then, the liver tissues of mice were excised for the detection of liver metastasis. As presented in Figure 4E, both YQ456 treatment and sgMYOF treatment decreased the numbers and fluorescence intensities of liver nodules. Although sufficient evidence is not available, these findings elucidated that YQ456 may suppress the metastatic propensity of colorectal cancer *in vivo*.

### 3.6 YQ456 hinders colorectal cancer development in a PDX xenograft mouse model

**FIGURE 5:**
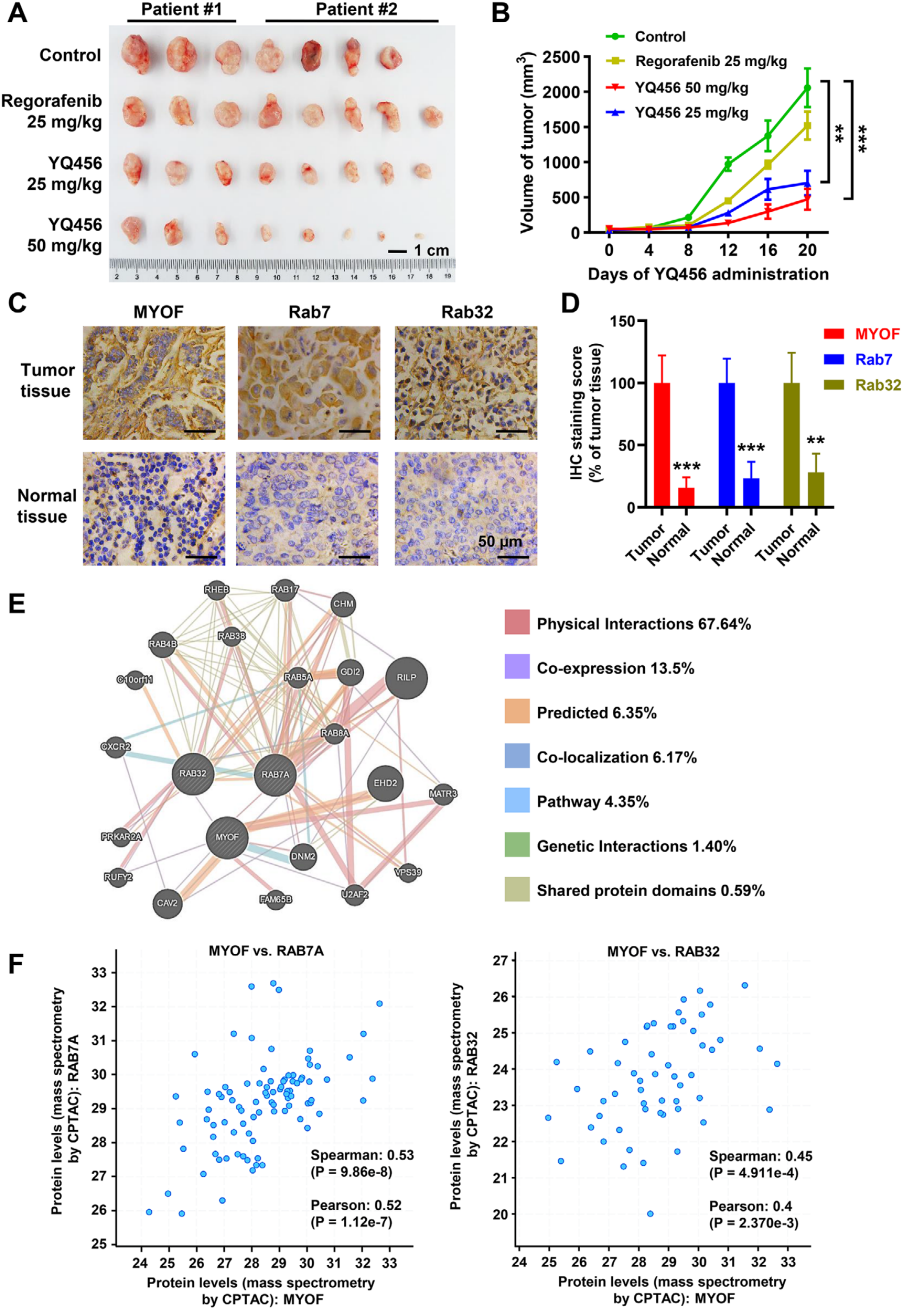
YQ456 prevents the development of colorectal cancer in the PDX mouse model. A, The tumor morphology image of mice in each group. Scale bars, 1 cm. B, The statistical analysis of tumor volume in each group. C, The staining signals (brown) of MYOF, Rab7, and Rab32 in tumor tissues and adjacent normal tissues. Nuclei were counterstained with DAPI (blue). Scale bars, 50 μm. D, The quantification of IHC staining. E, The interaction network of MYOF in the GeneMANIA database. F, The co‐expression relationships between MYOF and Rab7A, and between MYOF and Rab32 in tumor tissues from colorectal cancer patients. Data are presented as mean ± SD. **P < .01; ***P < .001

Patient‐derived tumor tissues were subcutaneously inoculated into BALB/c nude mice to establish a PDX mouse model of colorectal cancer. Interestingly, YQ456 displayed a significantly higher inhibition on colorectal cancer growth than regorafenib at the same dose (Figure 5A,B). Notably, no weight loss of mice was detected in all groups (data not shown). To further evaluate the clinical therapeutic potential of YQ456, two patient‐derived cell lines (#1 and #2) were established derived from the tumor tissues of two colorectal cancer patients (#1 and #2), respectively. The results indicated that YQ456 markedly suppressed the cell proliferation (Figure S6A), clone formation (Figure S6B), and cell invasion (Figure S6C) of the patient‐derived cell lines. Overall, YQ456 exhibits excellent antitumor effects in the colorectal cancer PDX model.

### 3.7 The functional interactions between MYOF and Rabs

Previous studies suggested that MYOF and Rabs have similar phenotypic and functional characteristics in vesicle trafficking.^13^, ^26^ The expression levels of MYOF, Rab7, and Rab32 in tumor tissues were significantly higher than those in normal tissues, implying the co‐expression relationships between MYOF and Rab7, and between MYOF and Rab32 (Figure 5C,D). Moreover, the functional protein‐protein interaction network of MYOF in the GeneMANIA database displayed the protein interaction between MYOF and Rab7A, as well as a previously unreported co‐expression between MYOF and Rab32 (Figure 5E). Additionally, the analysis using the cBioPortal for Cancer Genomics database further confirmed the co‐expression relationships between MYOF and Rab7A, and between MYOF and Rab32 (Figure 5F). In summary, evidences from bioinformatics analyses and IHC assay validate the potential interactions between MYOF and Rabs.

### 3.8 YQ456 inhibits the interaction between MYOF and Rab7

**FIGURE 6:**
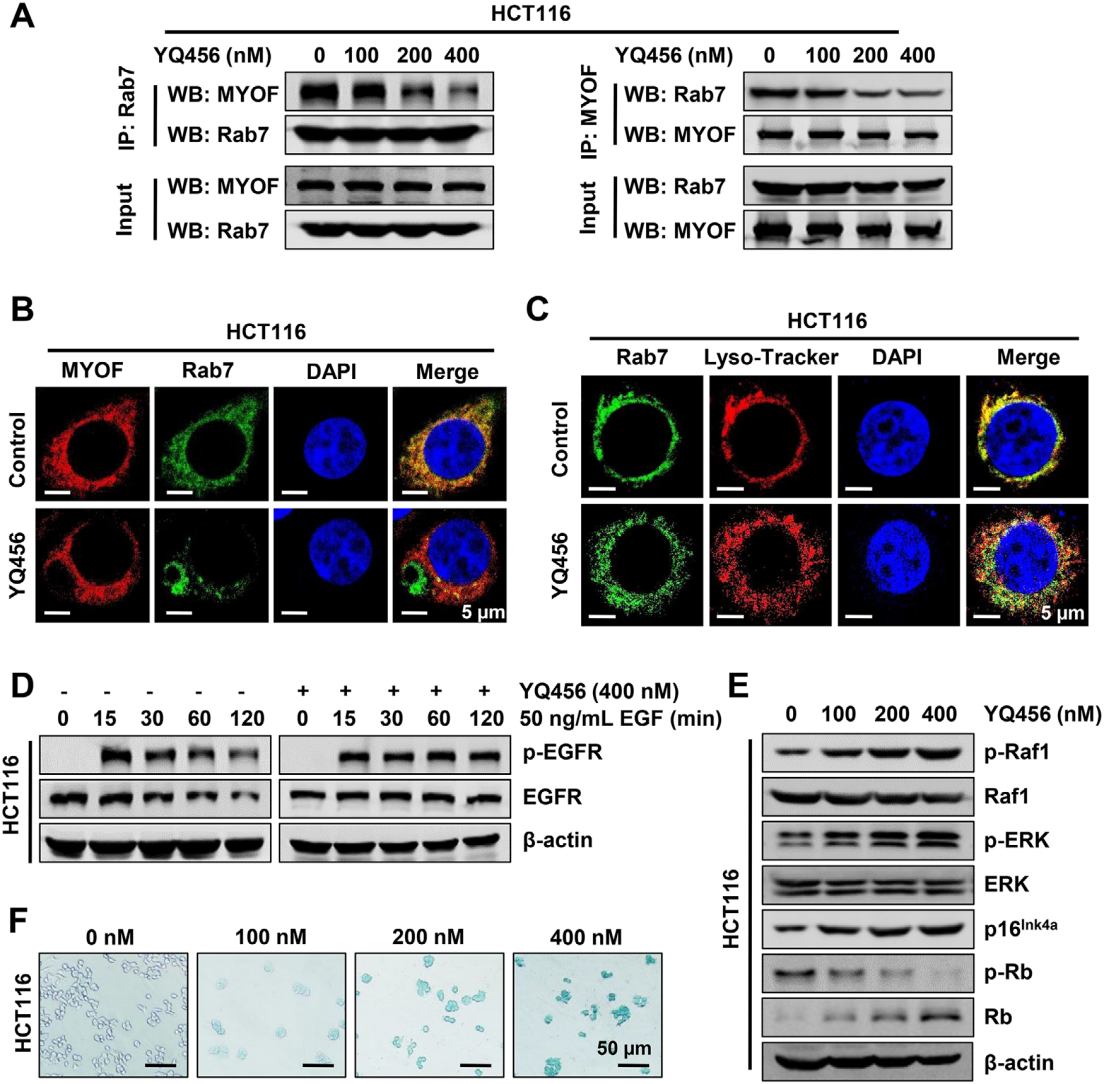
YQ456 disrupts the interaction between MYOF and Rab7 to halt intracellular transport. A, YQ456 suppressed the interaction between MYOF and Rab7 in HCT116 cells. B, The co‐localization of MYOF and Rab7 in HCT116 cells. C, YQ456 inhibited the lysosomal localization of Rab7 in HCT116 cells. D, YQ456 perturbed the degradation of EGF‐induced p‐EGFR in HCT116 cells. E, YQ456 promoted oncogene‐induced senescence in HCT116 cells. F, YQ456 induced the senescent phenotype of HCT116 cells in β‐galactosidase staining assay

Given the similar roles of MYOF and Rab7 in vesicular trafficking and their co‐localization in late endosome,^27^ we hypothesized that YQ456 may disrupt the interaction between MYOF and Rab7 to suppress late endosome‐related vesicle trafficking. In the co‐IP analysis, HA‐MYOF (Addgene, #22443) and Flag‐Rab7 plasmids were co‐transfected into HEK293T cells. Flag‐Rab7 protein was then immunoprecipitated with Flag M2 affinity gel (Sigma, A2220), and the immune‐captured complex was detected by anti‐HA antibody. The interaction between MYOF and Rab7 was dose‐dependently inhibited by YQ456 (Figure S7A). This finding was further confirmed using a reciprocal co‐IP assay by anti‐HA antibody‐conjugated agarose beads and anti‐Flag antibody (Figure S7A). Subsequently, a similar phenomenon in co‐IP assay was observed in HCT116 cells (Figure 6A). Besides, YQ456 significantly decreased the co‐localization of MYOF and Rab7 as detected by confocal laser scanning microscopy (CLSM) (Figure 6B). In addition, we evaluated the effect of YQ456 on the Rab7‐mediated fusion of late endosome and lysosome. As observed in the CLSM images, YQ456 significantly reduced the localization of Rab7 to lysosome membranes (indicated by Lyso‐Tracker) in HCT116 cells (Figure 6C). To sum up, YQ456 interferes with MYOF‐Rab7 complex formation and reduces the lysosomal localization of Rab7, thus synergistically disrupting late endosome‐lysosome fusion.

### 3.9 YQ456 impedes Rab7‐dependent intracellular transport

Emerging evidence suggested that MYOF promotes tumor progression by enhancing the stability of RTKs.^28^ Furthermore, Rab7 promotes EGFR degradation by mediating late endosome‐lysosome fusion.^29^ Therefore, the role of YQ456 in RTK phosphorylation was evaluated using a Proteome Profiler Human Phospho‐RTK Array Kit. The phosphorylation levels of several RTKs (indicated by red boxes) in YQ456‐treated HCT116 cells were significantly lower than those in HCT116 cells (Figure S7B and C). A similar effect of MYOF depletion on RTK activity has been confirmed in previous studies.^11^, ^30^ Notably, EGFR distinguishes itself from these other RTKs in that it is modulated by receptor down‐regulation.^30^ As illustrated in Figure 6D,E, YQ456 promoted p‐EGFR stability in cancer cells, thereby increasing the aberrant activation of EGFR pathway, leading to oncogene‐induced senescence (OIS), as evidenced by increased p16^Ink4a^ expression and reduced p‐Rb expression. Additionally, MYOF silencing promoted the separation of caveolin from internalized EGFR, thus escaping the lysosomal degradation of EGFR (Figure S7D‐F). Subsequently, the senescence‐inducing effects of YQ456 treatment (Figure 6F) and MYOF knockout treatment (Figure S7G) were further confirmed by senescence‐associated β‐galactosidase analysis. In summary, YQ456 inhibits Rab7‐dependent vesicle trafficking and mediates intracellular signaling pathways by targeting MYOF.

### 3.10 YQ456 prevents Rab7‐dependent exosome secretion by targeting MYOF

**FIGURE 7:**
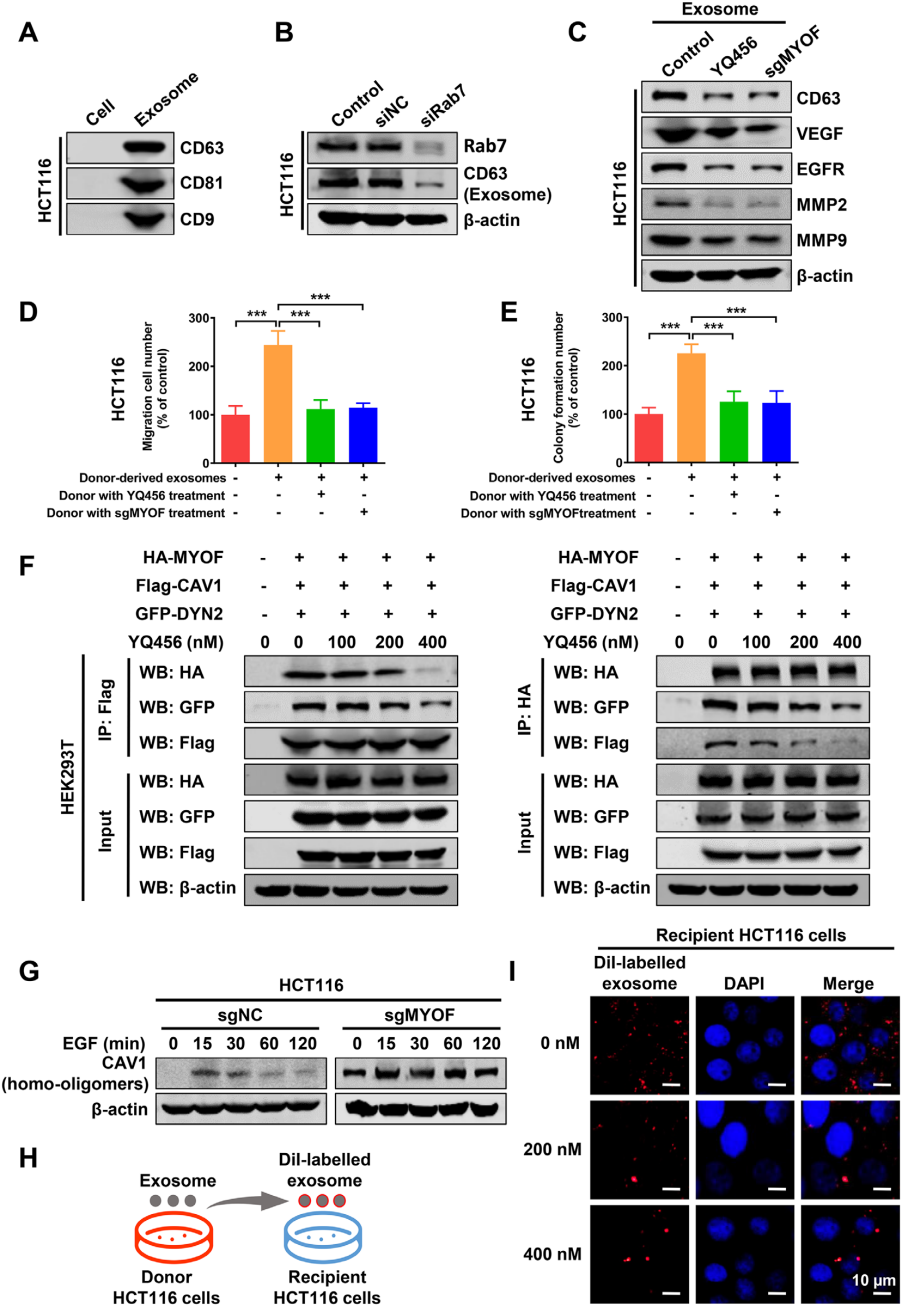
YQ456 prevents Rab7‐dependent exosome secretion and internalization by targeting MYOF. A, The expression of specific exosome markers CD63, CD81, and CD9 was detected in the purified exosomes. B, Rab7 knockdown decreases the exosome secretion of HCT116 cells. C, YQ456 reduced the expression of proliferation and migration‐related proteins in exosomes. D, The pro‐invasion ability of exosomes was significantly lower in the YQ456 group and sgMYOF group than that in the sgNC group. E, Both YQ456 treatment and sgMYOF treatment in HCT116 cells decreased the proliferation‐promoting capacity of secretory exosomes. F, YQ456 disrupted the trimeric complex formation in HEK293T cells as shown by co‐IP assay. G, MYOF depletion increased CAV1 oligomers in HCT116 cells with EGF induction. H, The schematic diagram of exosomes labeled with Dil. I, The exosome internalization was significantly lower in HCT116 cells with YQ456 administration. Data are presented as mean ± SD. ***P < .001

Another study unveiled that MYOF is highly relevant to endocytosis and cell membrane fusion.^9^ However, the specific molecular mechanism of MYOF on exosomes has not been reported. Additionally, Rab7 promotes exosome secretion of cancer cells to accelerate tumor progression.^31^ Therefore, we hypothesized that MYOF may modulate exosome secretion of colorectal cancer cells by disturbing the regulatory function of Rab7. As shown in Figure 7A, the exosomes derived from HCT116 cells were identified by the specific marker proteins (CD9, CD63, and CD81). Consistent with the result of siRab7 (Figure 7B), both YQ456 treatment and sgMYOF treatment remarkably reduced the exosome secretion, as evidenced by the decreased expression of specific marker protein CD63 (Figures 7B,C). Correspondingly, both YQ456 treatment and sgMYOF treatment significantly decreased the growth factors and matrix metalloproteases in exosomes (Figure 7C). Besides, the pretreatment of YQ456 or sgMYOF in donor HCT116 cells inhibited the tumor‐promoting effects of exosomes in recipient HCT116 cells (Figures 7D,E).

### 3.11 YQ456 targets MYOF to prevent exosome internalization

MYOF forms a complex with caveolin 1 (CAV1) and dynamin 2 (DYN2) to modulate RTK internalization.^32^ Further, CAV1 and DYN2 significantly promote exosome internalization.^33^, ^34^ Therefore, we explored the effect of YQ456 on exosome internalization. After co‐transfection with HA‐MYOF, Flag‐CAV1, and GFP‐DYN2 plasmids in HEK293T cells, cell lysates were subjected to immunoblotting in co‐IP assay. Interestingly, YQ456 dose‐dependently inhibited the formation of MYOF‐CAV1‐DYN2 tripartite complex (Figure 7F). Moreover, CAV1 formed inactive homo‐oligomers in HCT116 cells with MYOF depletion, implying the inactivation of MYOF‐CAV1‐DYN2 tripartite complex (Figure 7G).

Concomitant with the impairment of this complex, YQ456 reduced the internalization efficiency of DiI‐labelled exosomes into recipient HCT116 cells (Figures 7H,I; Figure S8A). However, the precise internalization pattern of exosomes in colorectal cancer remains controversial.^35^ To explore the molecular mechanism of exosome internalization, a series of internalization inhibitors were used to selectively suppress different internalization pathways. Both the CAV1 inhibitor (Nystatin) and DYN2 inhibitor (dynasore) markedly reduced the internalization efficiency of exosomes, whereas the clathrin inhibitor (chlorpromazine) exerted little inhibitory activity (Figure S8B,C). Therefore, YQ456 targets MYOF to prevent exosome secretion and exosome internalization, thereby suppressing the proliferation and invasion capacities of colorectal cancer cells.

### 3.12 YQ456 suppresses Rab32‐dependent mitochondrial metabolism and dynamics by targeting MYOF

**FIGURE 8:**
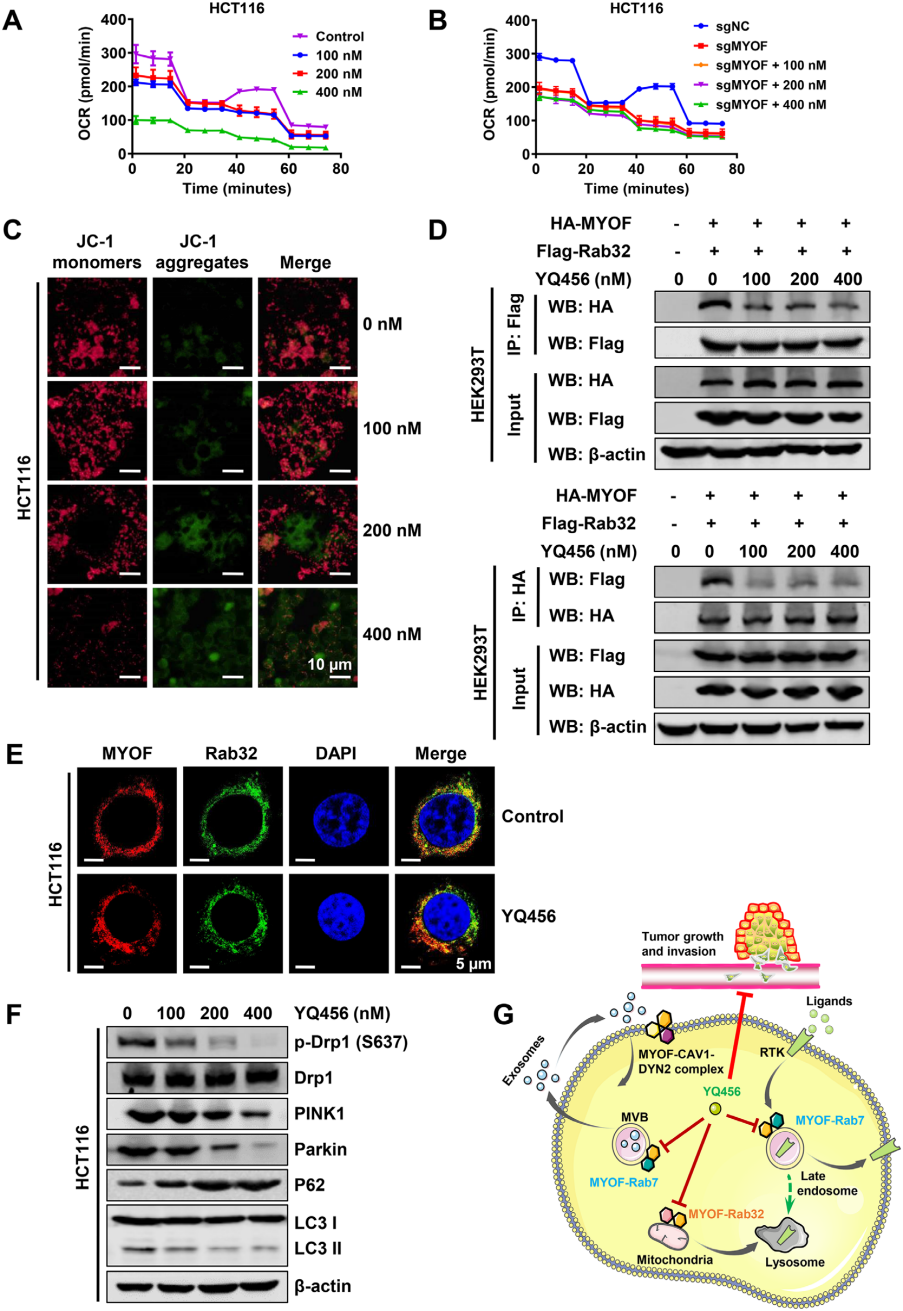
YQ456 targets MYOF to inhibit Rab32‐dependent mitochondrial metabolism. A, The mitochondrial OCR was measured using a Seahorse XFe96 analyzer to determine the effect of YQ456 on mitochondrial metabolism. B, The inhibition effect of YQ456 on mitochondrial OCR was weakened in sgMYOF HCT116 cells. C, YQ456 dose‐dependently reduced the MMP (Red) of HCT116 cells. Scale bars, 10 μm. D, The effect of YQ456 on the interaction between MYOF and Rab32 in co‐IP analysis. E, The effect of YQ456 on the co‐localization of MYOF (Red) and Rab32 (Green) as shown by IF analysis. Nuclei were counterstained with DAPI (blue). Scale bars, 5 μm. F, The expression of p‐Drp1 (S637), PINK1, Parkin, LC3‐II/LC3‐I, and p62 in HCT116 cells with YQ456 treatment. G, The schematic diagram illustrating the anti‐cancer mechanisms of YQ456 in colorectal cancer

Notably, Rab32 is the only Rab GTPase that participates in mitochondrial dynamics by inducing the phosphorylation of Drp1 at S637, an inhibitory phosphorylation site.^20^ Although the relationship between MYOF and Rab32 has not been reported, there are similar phenotypes and functional characteristics of mitochondrion in siMYOF cells and siRab32 cells. This prompted us to investigate whether YQ456 targeted MYOF to modulate mitochondrial metabolism and mitochondrial dynamics through Rab32‐related pathway.^15^, ^22^ The Seahorse assay showed that YQ456 significantly inhibited mitochondrial energy metabolism in HCT116 cells (Figure 8A). Additionally, this phenomenon was consistent with the results detected in sgMYOF cells (Figure S9A) and Rab32 knockdown cells (Figure S9B). Interestingly, YQ456 exerted no effect on mitochondrial OCR in MYOF knockout cells (Figure 8B). In contrast, YQ456 further reduced the mitochondrial OCR of HCT116 cells with Rab32 silencing (Figure S9B), implying that YQ456 affected mitochondrial function by targeting MYOF rather than Rab32. Besides, YQ456 reduced mitochondrial membrane potential (MMP; Figure 8C) and increased mitochondrial ROS production (Figure S9C) in HCT116 cells. These results were consistent with the phenomenon observed in sgMYOF cells (Figure S9D,E).

To explore the potential relationship between MYOF and Rab32, HEK293T cells were transfected with HA‐MYOF and Flag‐Rab32 plasmids in co‐IP assay. The cell lysate was incubated with antibody‐conjugated agarose beads, followed by immunoblotting with corresponding antibodies. As presented in Figure 8D, YQ456 dose‐dependently hindered the formation of MYOF‐Rab32 complex. Besides, the IF assay showed that YQ456 markedly decreased the co‐localization of MYOF and Rab32 in HCT116 cells (Figure 8E). A previous study explored that Rab32 prevents mitochondrial fission by promoting the phosphorylation of Drp1 at S637.^22^ As shown in Figure S9F, YQ456 interfered with the localization of Rab32 to the mitochondrion membrane. Furthermore, YQ456 dose‐dependently inhibited Drp1 phosphorylation at S637 in HCT116 cells (Figure 8F), thereby leading to continuous mitochondrial fission. Additionally, a similar phenomenon was detected in HCT116 cells with siRab32 treatment or sgMYOF treatment (Figure S9G,H). Rab32 silencing causes the structural destruction of mitochondrion and induced mitochondrial fragmentation, thus promoting cancer cell death. In this case, cancer cells will trigger the autophagy‐lysosomal degradation of mitochondrion to maintain mitochondrial homeostasis and escape programmed cell death.^36^ Interestingly, YQ456 disrupted mitochondrial autophagy to induce cancer cell death by inhibiting Rab7‐meditated lysosomal degradation, as evidenced by the decline of PINK1/Parkin pathway, decreased LC3‐II/LC3‐I ratio, and elevated P62 expression (Figure 8F). Therefore, YQ456 affects multiple functions of Rab7 and Rab32 by targeting MYOF, thereby preventing colorectal cancer progression. A schematic diagram of the molecular mechanisms of YQ456 was depicted in Figure 8G.

### 3.13 The anticancer activities of YQ456 are positively correlated with MYOF expression level

LoVo cells with MYOF knockout (sgMYOF LoVo cells) were constructed using the CRISPR/Cas9 system to analyze the off‐target effects of YQ456 (Figure S10A). Interestingly, YQ456 displayed no effects on EMT reversion and RTK modulation in sgMYOF LoVo cells (Figure S10B–D). The weakened anti‐cancer capabilities of YQ456 in sgMYOF cells was consistent with the above results (Figure S10E,F). Notably, the overexpression of MYOF in sgMYOF cells rescued the anti‐cancer activities of YQ456 (Figure S10E,F). On the contrary, no significant correlation existed between the expression level of MYOF and the anti‐cancer activities of lead compound WJ460 (data not shown). Although the two MYOF inhibitors have comparable anti‐invasion activities (Figure 1C), evidences from the binding affinity assay (Figure 1C) and target identification experiments (Figure S10) suggested that WJ460 may have other important targets in addition to MYOF, while YQ456 has a higher targeting specificity to MYOF in colorectal cancer cells.

Further, multiple cell lines with different expression levels of MYOF were selected from the Cancer Cell Line Encyclopedia database (Table S3). The expression levels of MYOF in cancer cells were significantly higher than those in normal cells (Figure S10G). Moreover, the anti‐cancer activities of YQ456 were positively correlated with MYOF expression level in transwell invasion assay (Figure S10H). On the whole, these findings imply that YQ456 is a potential therapeutic agent in MYOF‐driven cancers.

## 4 DISCUSSION

The excessive activation of vesicle trafficking is essential for the modulation of signaling pathways and biochemical processes of organelles in cancer cells.^37^ Aberrant vesicle trafficking, the pathological hallmark of many malignancies, has recently attracted increasing attention. Although both MYOF and Rabs exert essential roles in regulating vesicle trafficking, their relationships have not been clarified. Recent work suggested MYOF localizes in Rab7‐positive late endosomes and regulates receptor transport in the endolysosomal system.^27^ Besides, previous immunoprecipitate proteomic analysis demonstrated that Rab GTPase effector Rab‐coupling protein forms a complex with MYOF to regulate RTK trafficking, thus promoting cancer metastasis.^38^ Although evidence is insufficient yet, MYOF and Rabs may synergistically promote vesicular trafficking in cancer cells.

Our previous investigation found that WJ460 sequesters MYOF from Rab7‐positive late endosome, thereby inhibiting the biological activity of endosomal system in breast cancer cells.^6^ However, another study unveiled that Rab7 expression decreases upon MYOF knockdown,^39^ implying the relationship between MYOF and Rab7 is more complicated than that in our previous report. In the present study, we further explored that MYOF inhibition suppressed the formation of MYOF‐Rab7 complex and the co‐localization of Rab7 to the late endosome in colorectal cancer cells. The impairment of MYOF‐Rab7 complex interfered with receptor protein degradation, exosome secretion, and mitochondrial autophagy. Besides, we revealed the formation of MYOF‐Rab32 complex and the co‐localization of MYOF and Rab32 in mitochondria. However, further investigation into the crystal structure of MYOF is warranted to explore the relationships between MYOF and Rab family proteins.

Existing evidence has suggested that MYOF is involved in the secretion of endothelial cells and the membrane fusion of myoblast.^40^ Previous studies on MYOF mainly explore its roles in intracellular signaling regulation and mitochondrial metabolism in cancer cells. However, few reports pay attention to the roles of MYOF in endocytosis and exocytosis in cancer cells. Cancer cell‐derived exosomes are internalized into recipient cells to promote cancer initiation and progression.^41^ The present study unveiled that MYOF inhibition prevents the secretion and internalization of exosomes in colorectal cancer cells. These findings are contrary to that exosome secretion is unaffected by MYOF inhibition in breast and pancreatic cancers.^39^ However, our data retrieved from the cBioPortal for Cancer Genomics database presented a significant positive relationship between MYOF and Rab7A in colorectal cancer. In contrast, this correlation does not exist in breast and pancreatic cancers. Therefore, the different conclusions of exosome secretion in these cancer types can be attributed to tumor intrinsic heterogeneity.

Although controversy lies in the effect of MYOF on cancer cell proliferation, most studies have proved that MYOF knockdown inhibits cancer cell migration and metastasis. We proved that targeting MYOF prevents the cell invasion of colorectal cancer. Besides, the *in vivo* anti‐growth effect of YQ456 may be attributed to the inhibition of both angiogenesis and exosome secretion.

In this study, YQ456 targeting MYOF exhibits promising prospects for clinical application. Currently, VEGF and EGFR are the most important therapeutic targets of colorectal cancer. Given the significant roles of YQ456 in the modulation of RTK pathways, YQ456 may act synergistically with VEGF inhibitors and EGFR inhibitors. Despite plenty of fundamental work remains to be done, YQ456 targeting MYOF possibly provides therapeutic benefits to patients with VEGF inhibitor resistance or EGFR inhibitor resistance. Moreover, based on our experimental findings, YQ456 may alert the innate immune system by inducing senescence, a non‐lethal form of growth arrest. The protective senescence‐related immune surveillance triggered by YQ456 can be a potential tumor‐suppressive program in clinical anti‐cancer treatments. However, further research should be undertaken to validate this hypothesis.

Although sufficient confirmatory experiments have been done, the present study has several methodological weaknesses. For example, a genetic mouse model with intrinsic metastasis development will facilitate the functional exploration of MYOF in the spontaneous metastasis of colorectal cancer. Besides, an orthotopic metastasis PDX model of colorectal cancer is required to further validate the *in vivo* anti‐growth and anti‐metastasis efficacies of YQ456.

In summary, we have identified a novel potent and selective inhibitor of MYOF to treat colorectal cancer with significant anti‐tumor activities and low toxicity. Additionally, this study provides *in vivo* evidence that the expression level of MYOF is proportional to the invasive capacity of colorectal cancer cells. We also reveal that YQ456 disturbs the previously unreported cross‐talks between MYOF and Rab7, and between MYOF and Rab32. Impressively, the excellent therapeutic efficacy of YQ456 in a PDX mouse model implies a promising clinical therapeutic prospect of targeting MYOF in colorectal cancer.

## AUTHOR CONTRIBUTIONS

Yuan He, Zhengfang Yi, Yihua Chen, and Mingyao Liu conceived the idea and designed the study. Yuan He performed most experiments and analyzed data. Weiqiong Kan, Yun Hao, Anling Huang, Minna Wang, and Qingqing Wang assisted with biological experiments and statistical analysis. Yunqi Li, and Haijun Gu helped in the synthesis of compounds. Jinlian Chen and Zhenliang Sun contributed to manuscript preparation. All authors read and approved the final manuscript.

## CONFLICT OF INTEREST

The authors have declared that no conflict of interest exists.

## ETHICS APPROVAL AND CONSENT TO PARTICIPATE

Tissue samples were obtained from two colorectal cancer patients in Southern Medical University Affiliated Fengxian Hospital (patient #1) and Renji Hospital Affiliated to Shanghai Jiaotong University School of Medicine (patient #2), respectively. Informed consents were obtained from the patients. All animal experimental protocols and ethical guidelines were approved by the Animal Investigation Committee of the Institute of Biomedical Sciences, East China Normal University.

## Supporting information

SUPPORTING INFORMATION

SUPPORTING INFORMATION

SUPPORTING INFORMATION

SUPPORTING INFORMATION

SUPPORTING INFORMATION

SUPPORTING INFORMATION

SUPPORTING INFORMATION

SUPPORTING INFORMATION

SUPPORTING INFORMATION

SUPPORTING INFORMATION

SUPPORTING INFORMATION

SUPPORTING INFORMATION

SUPPORTING INFORMATION

## Data Availability

The data used in the current study are available from the corresponding author on reasonable request.
