# Supplementary figures and images for: A potent and selective small molecule inhibitor of myoferlin attenuates colorectal cancer progression
Source: Clin Transl Med. 2021 Feb 7;11(2):e289. doi: 10.1002/ctm2.289 (PMC7868085; doi:10.1002/ctm2.289)

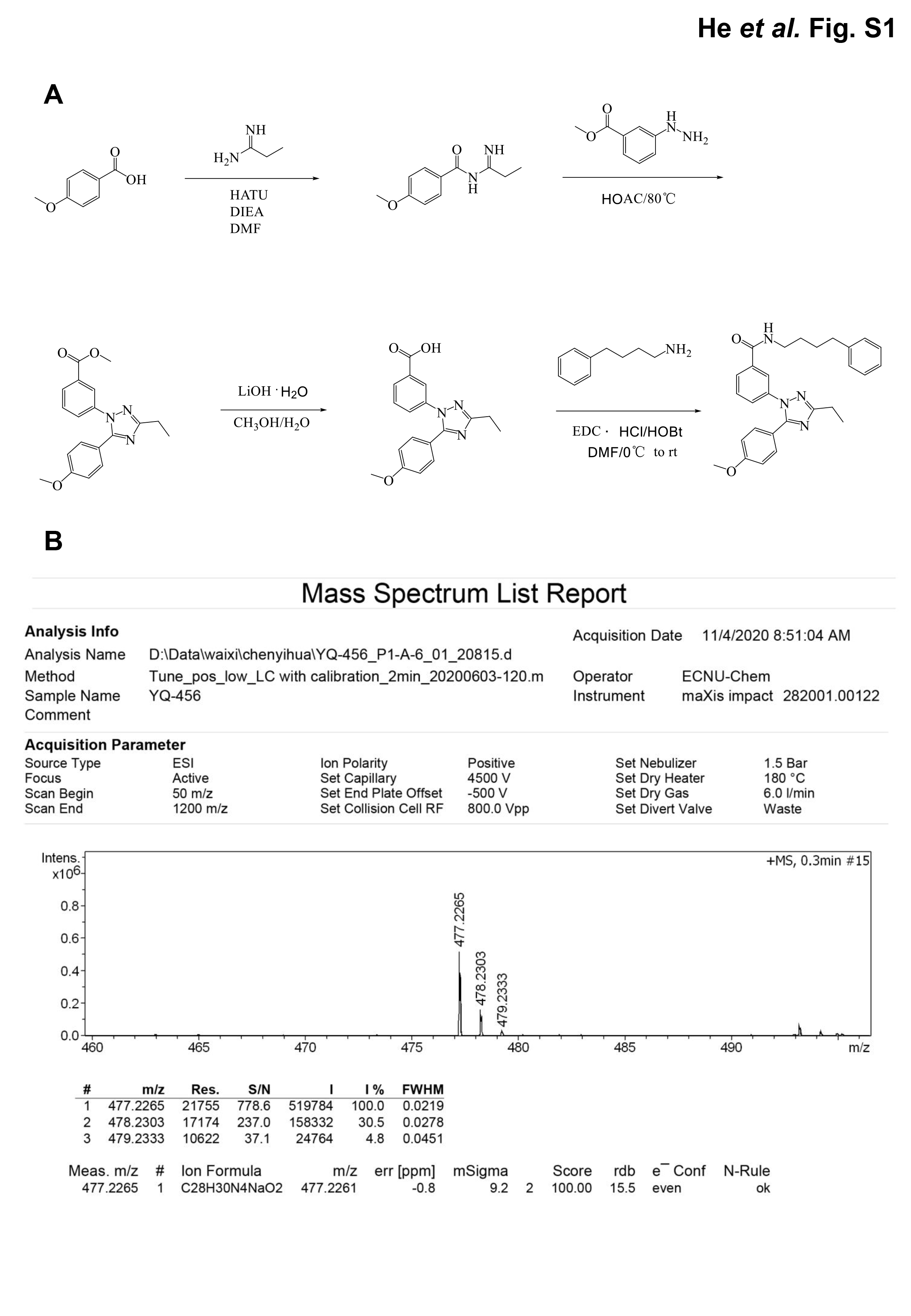

Supplement: Supplementary file 1 — SUPPORTING INFORMATION [file CTM2-11-e289-s001.tif]

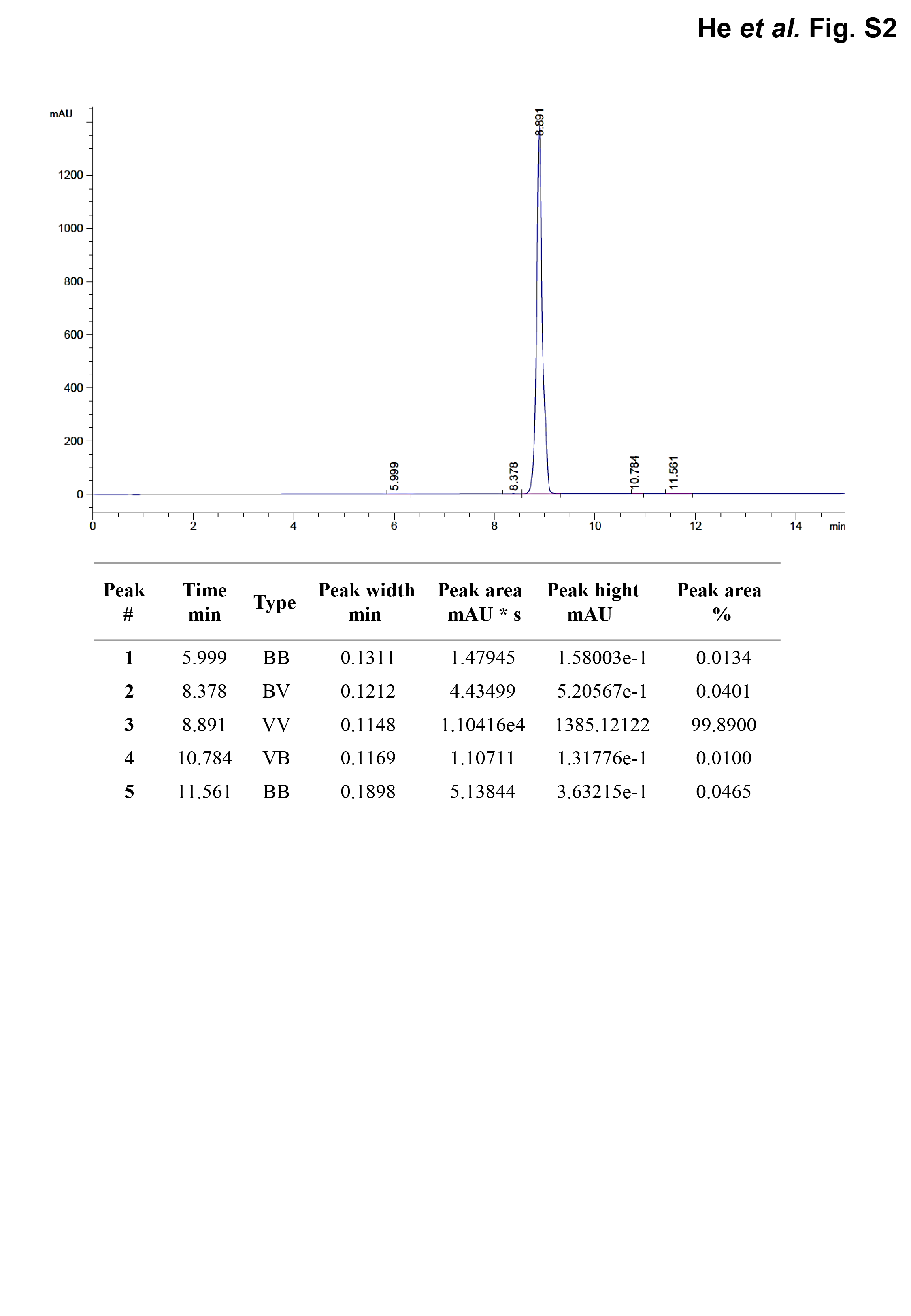

Supplement: Supplementary file 2 — SUPPORTING INFORMATION [file CTM2-11-e289-s002.tif]

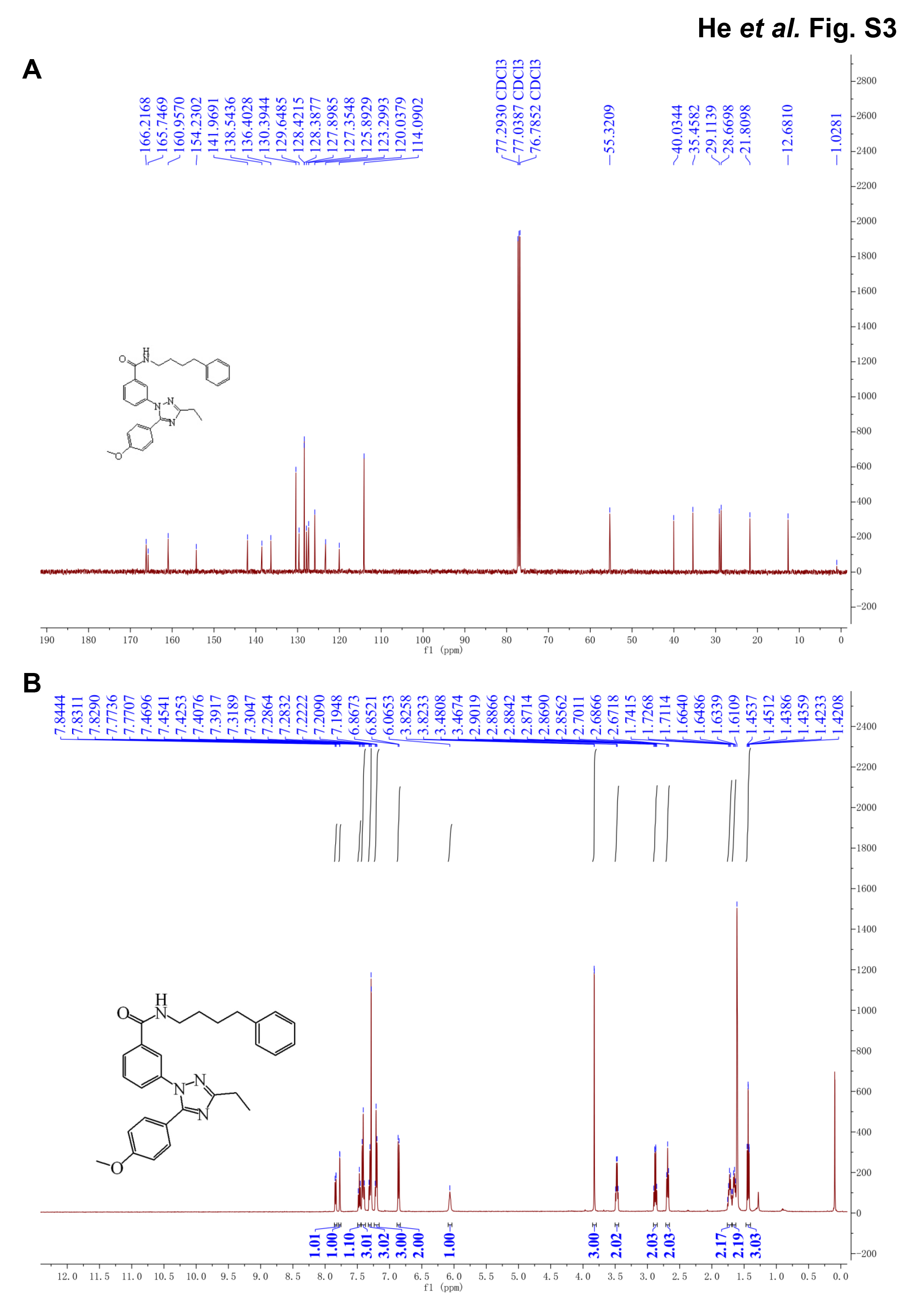

Supplement: Supplementary file 3 — SUPPORTING INFORMATION [file CTM2-11-e289-s003.tif]

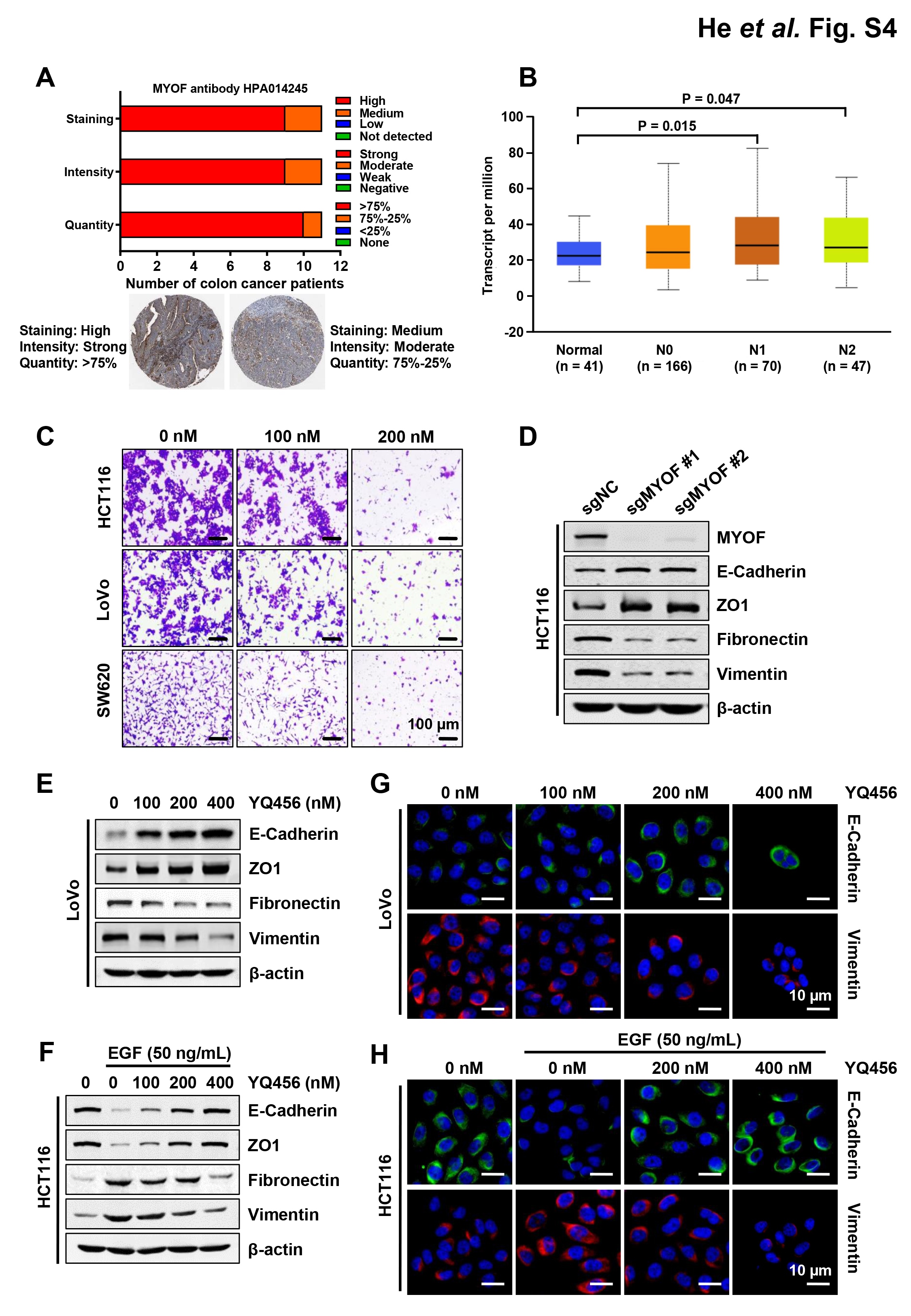

Supplement: Supplementary file 4 — SUPPORTING INFORMATION [file CTM2-11-e289-s004.tif]

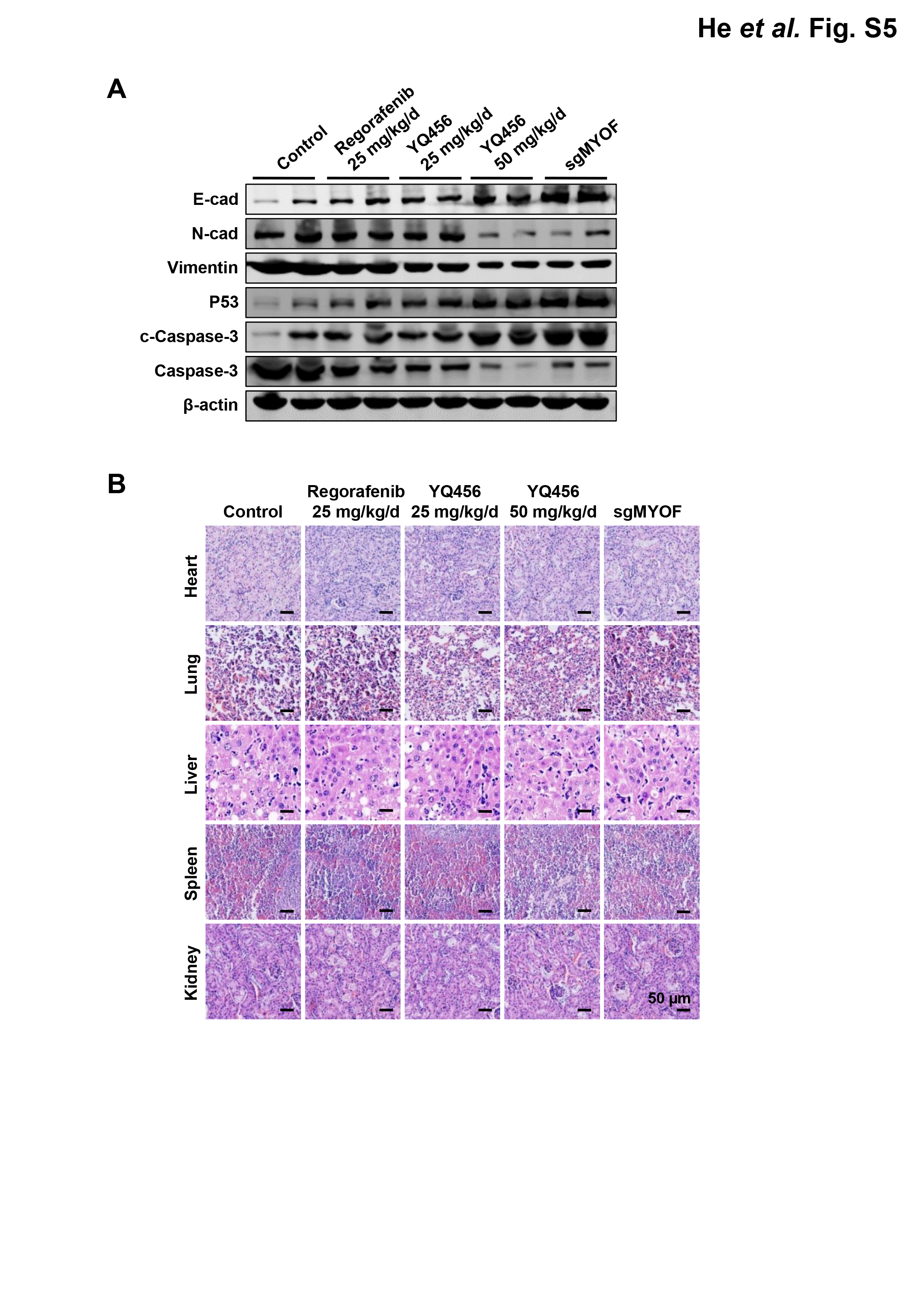

Supplement: Supplementary file 5 — SUPPORTING INFORMATION [file CTM2-11-e289-s005.tif]

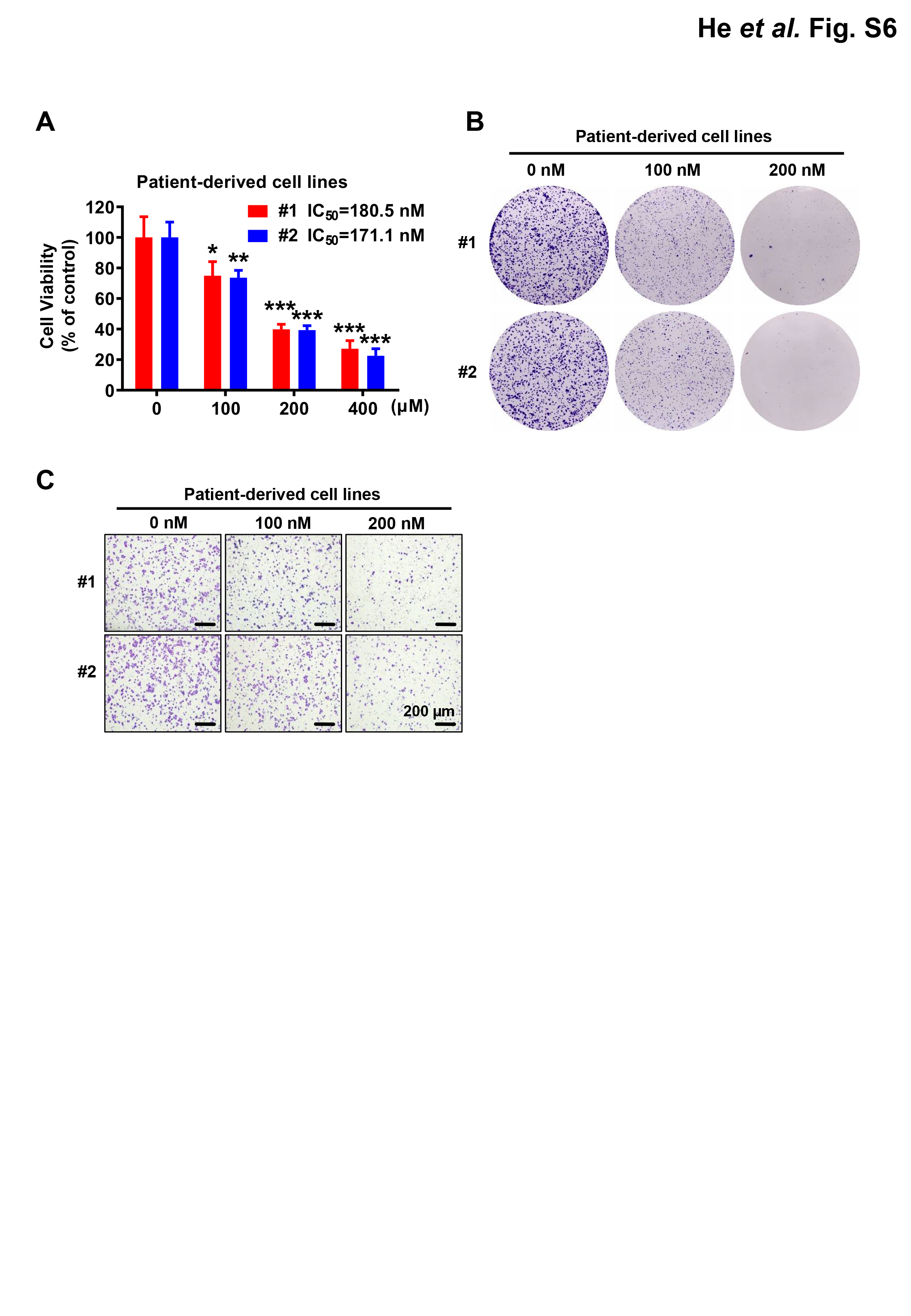

Supplement: Supplementary file 6 — SUPPORTING INFORMATION [file CTM2-11-e289-s006.tif]

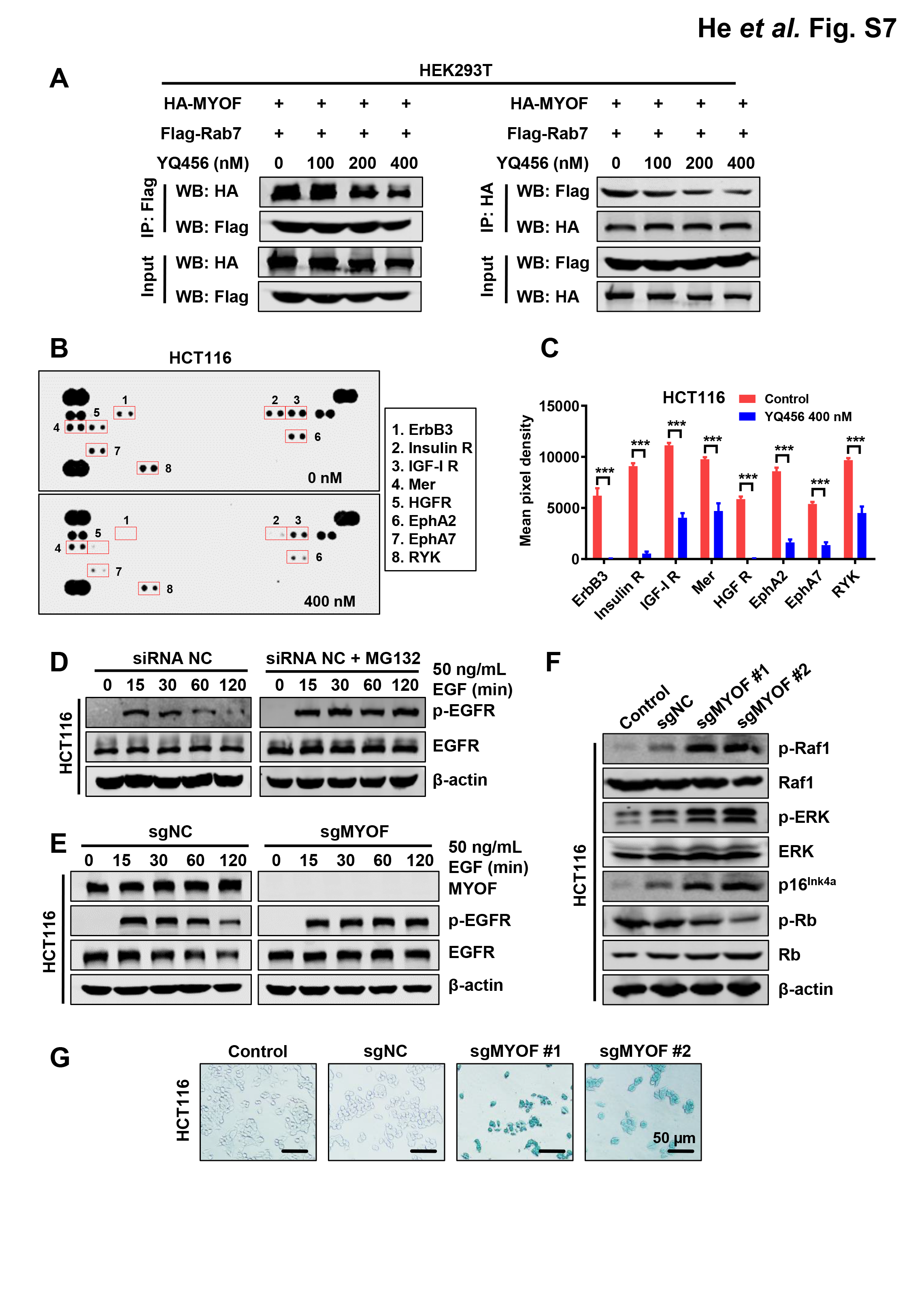

Supplement: Supplementary file 7 — SUPPORTING INFORMATION [file CTM2-11-e289-s007.tif]

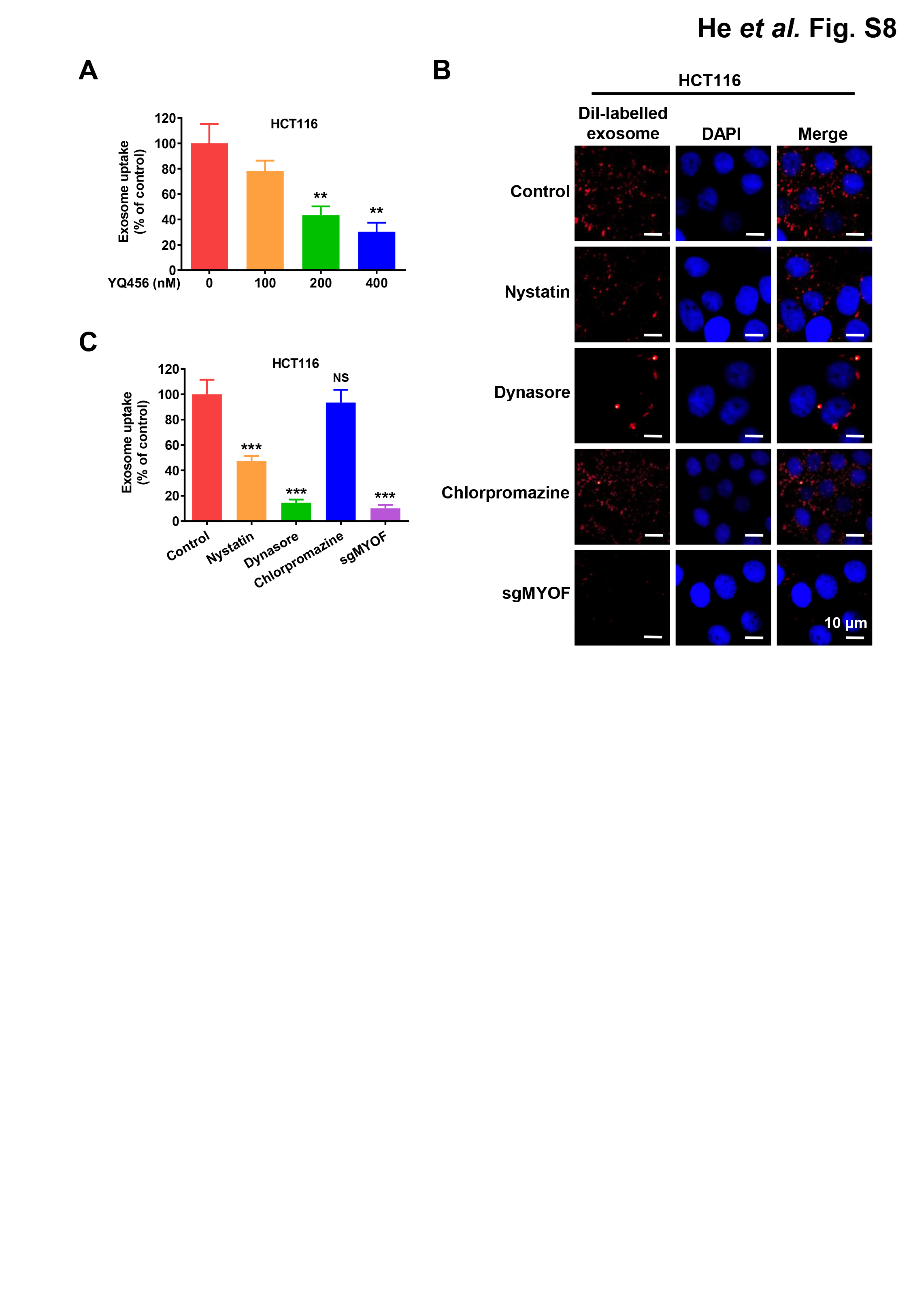

Supplement: Supplementary file 8 — SUPPORTING INFORMATION [file CTM2-11-e289-s008.tif]

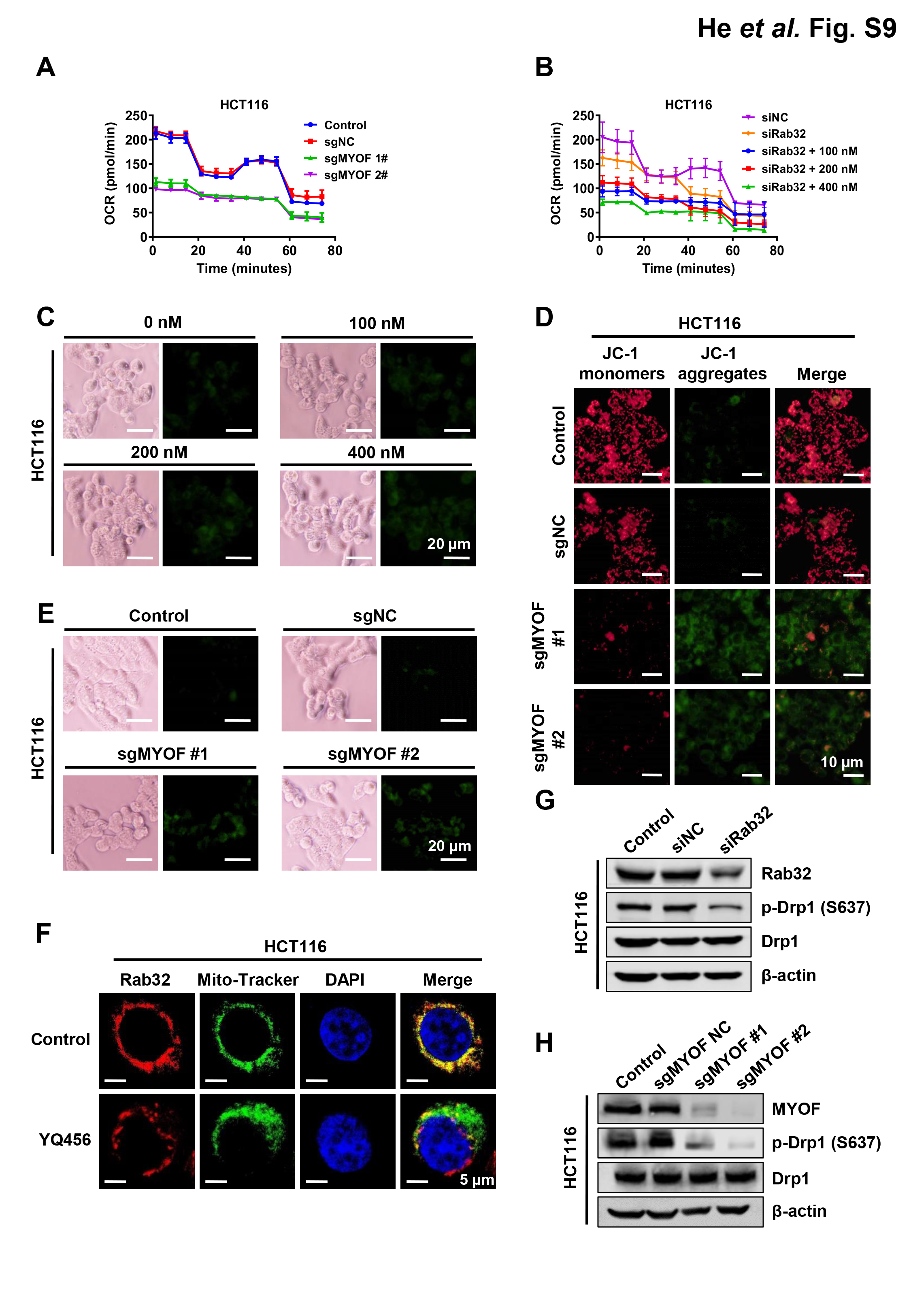

Supplement: Supplementary file 9 — SUPPORTING INFORMATION [file CTM2-11-e289-s009.tif]

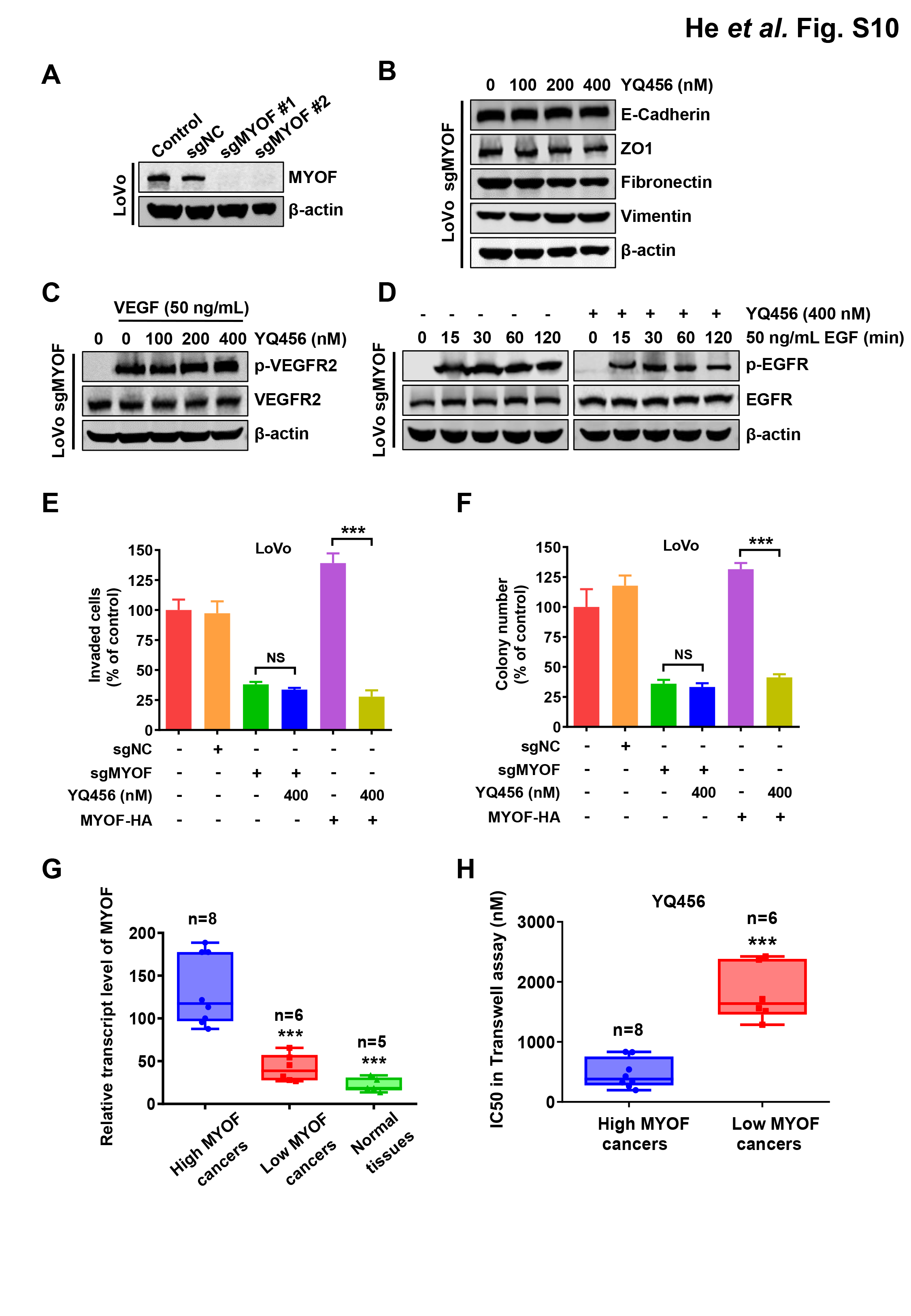

Supplement: Supplementary file 10 — SUPPORTING INFORMATION [file CTM2-11-e289-s010.tif]

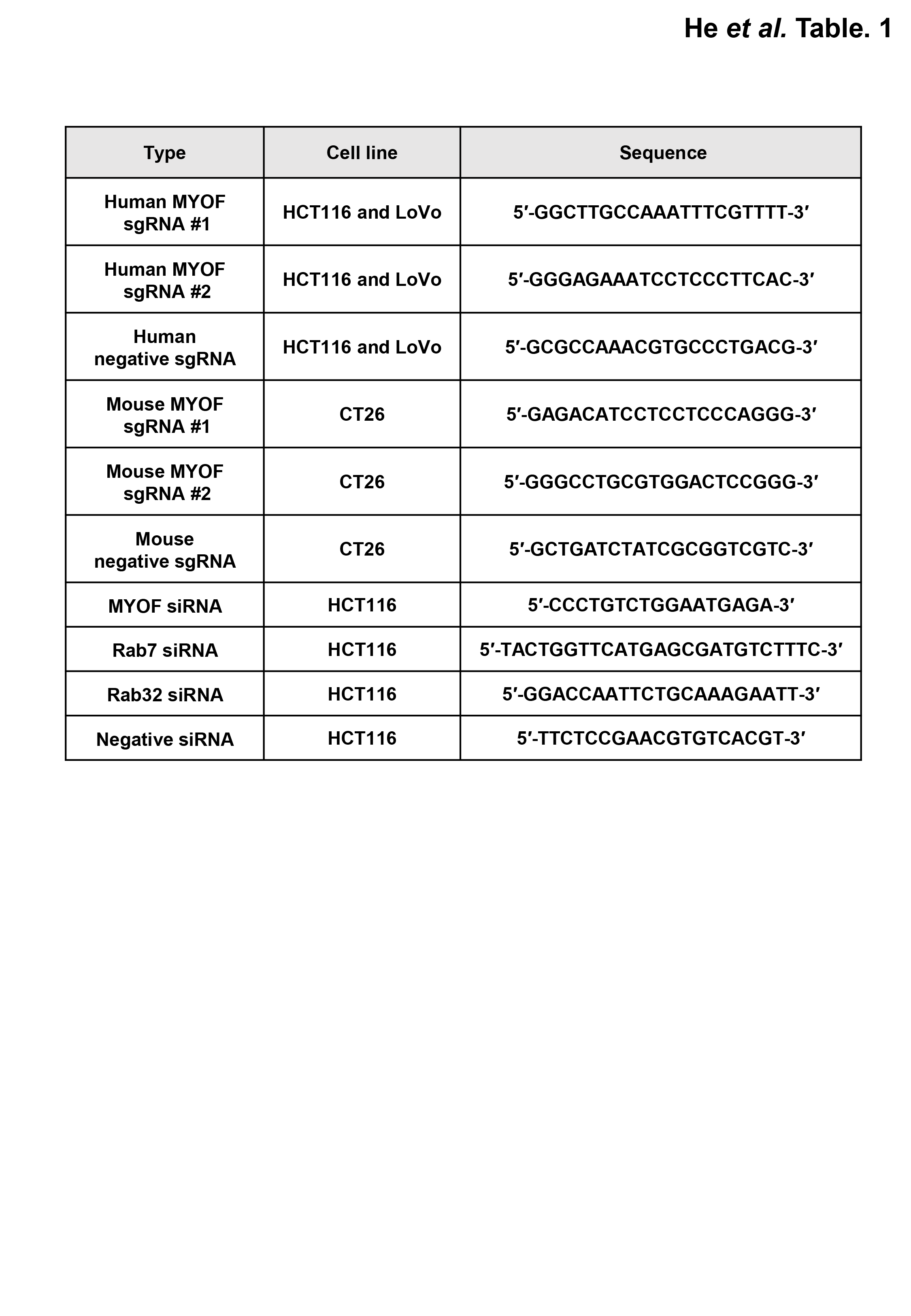

Supplement: Supplementary file 11 — SUPPORTING INFORMATION [file CTM2-11-e289-s011.tif]

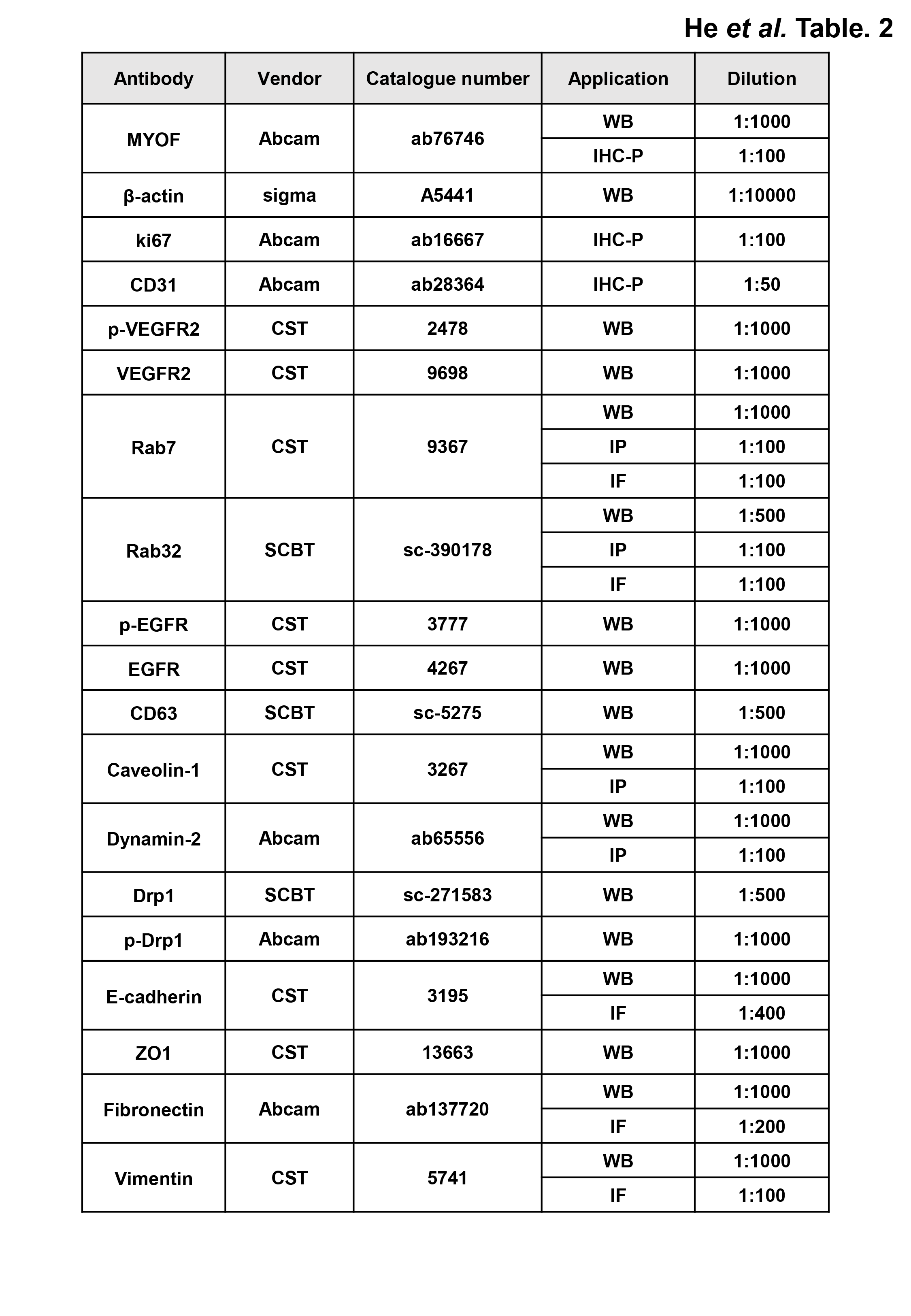

Supplement: Supplementary file 12 — SUPPORTING INFORMATION [file CTM2-11-e289-s012.tif]

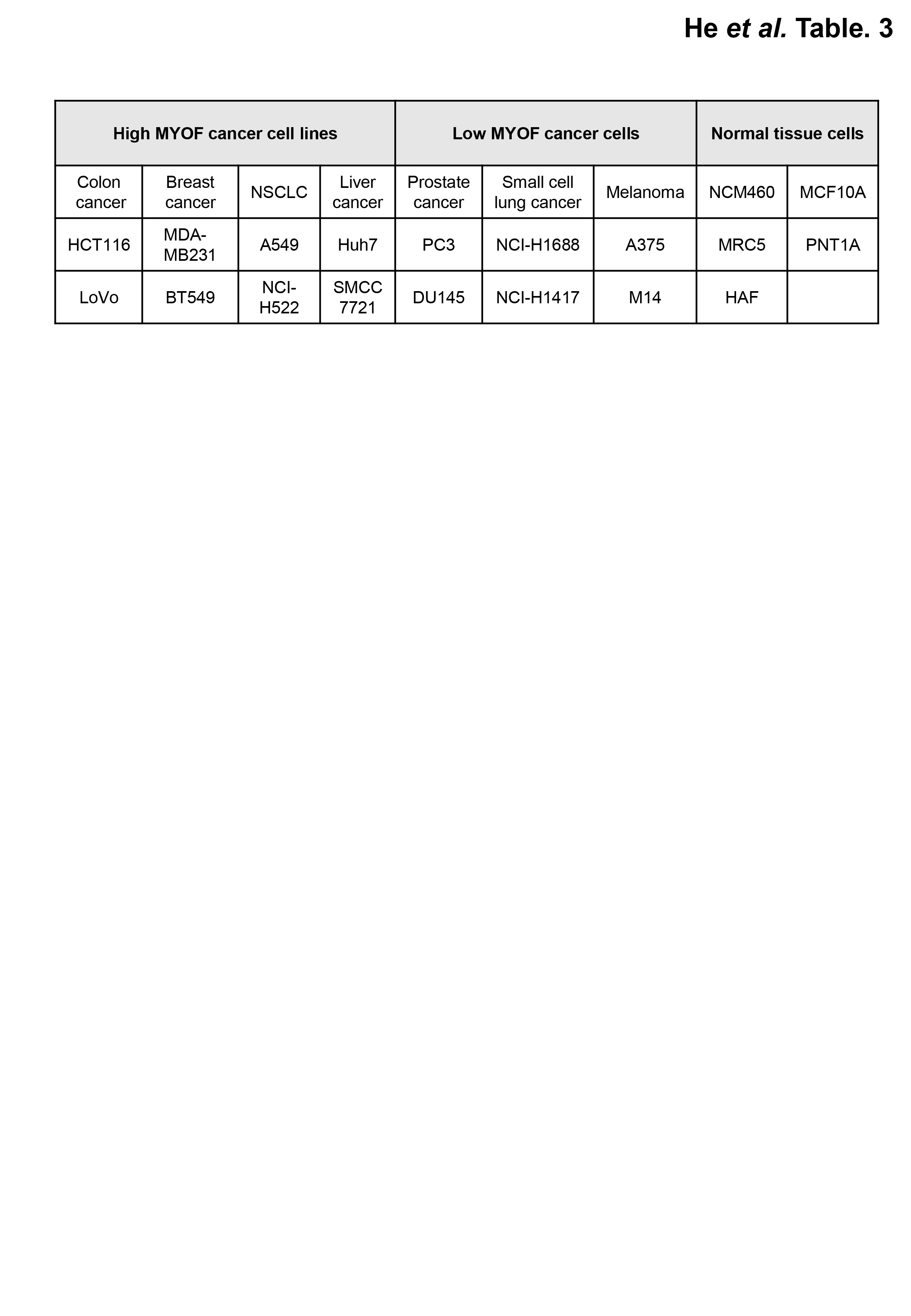

Supplement: Supplementary file 13 — SUPPORTING INFORMATION [file CTM2-11-e289-s013.tif]
